# Interleukin-1 alpha increases anti-tumor efficacy of cetuximab in head and neck squamous cell carcinoma

**DOI:** 10.1186/s40425-019-0550-z

**Published:** 2019-03-19

**Authors:** Madelyn Espinosa-Cotton, Samuel N. Rodman III, Kathleen A. Ross, Isaac J. Jensen, Kenley Sangodeyi-Miller, Ayana J. McLaren, Rachel A. Dahl, Katherine N. Gibson-Corley, Adam T. Koch, Yang-Xin Fu, Vladimir P. Badovinac, Douglas Laux, Balaji Narasimhan, Andrean L. Simons

**Affiliations:** 1 Free Radical and Radiation Biology Program, Department of Radiation Oncology, The University of Iowa, Iowa City, IA 52242 USA; 2 Holden Comprehensive Cancer Center, University of Iowa Hospitals and Clinics, Iowa City, IA 52242 USA; 3 Department of Chemical and Biological Engineering, College of Engineering, Iowa State University, Ames, IA 50011 USA; 4 Nanovaccine Institute, Iowa State University, Ames, IA 50011 USA; 5 Interdisciplinary Immunology Graduate Program, University of Iowa, Iowa City, IA 52242 USA; 6 Department of Pathology, University of Iowa, 1161 Medical Laboratories, Iowa City, IA 52242 USA; 7 San Jacinto College, Pasadena, TX 77505 USA; 8 Lincoln University, Lincoln, PA 19352 USA; 9 Department of Pathology, UT Southwestern Medical Center, Dallas, TX 75390 USA; 10 Department of Microbiology and Immunology, University of Iowa, Iowa City, IA 52242 USA; 11 Department of Internal Medicine - Hematology, Oncology and Blood and Marrow Transplantation, University of Iowa Hospitals and Clinics, Iowa City, IA 52242 USA

**Keywords:** EGFR, Cetuximab, Interleukin-1, Anakinra, Cytokines, Nanoparticle, HNSCC, Biomarker

## Abstract

**Background:**

Despite the high prevalence of epidermal growth factor receptor (EGFR) overexpression in head and neck squamous cell carcinomas (HNSCCs), incorporation of the EGFR inhibitor cetuximab into the clinical management of HNSCC has not led to significant changes in long-term survival outcomes. Therefore, the identification of novel therapeutic approaches to enhance the clinical efficacy of cetuximab could lead to improved long-term survival for HNSCC patients. Our previous work suggests that EGFR inhibition activates the interleukin-1 (IL-1) pathway via tumor release of IL-1 alpha (IL-1α), although the clinical implications of activating this pathway are unclear in the context of cetuximab therapy. Given the role of IL-1 signaling in anti-tumor immune response, we hypothesized that increases in IL-1α levels would enhance tumor response to cetuximab.

**Methods:**

Parental and stable myeloid differentiation primary response gene 88 (MyD88) and IL-1 receptor 1 (IL-1R1) knockdown HNSCC cell lines, an IL-1R antagonist (IL-1RA), neutralizing antibodies to IL-1α and IL-1β, and recombinant IL-1α and IL-1β were used to determine cytokine production (using ELISA) in response to cetuximab in vitro. IL-1 pathway modulation in mouse models was accomplished by administration of IL-1RA, stable overexpression of IL-1α in SQ20B cells, administration of rIL-1α, and administration of a polyanhydride nanoparticle formulation of IL-1α. CD4^+^ and CD8^+^ T cell-depleting antibodies were used to understand the contribution of T cell-dependent anti-tumor immune responses. Baseline serum levels of IL-1α were measured using ELISA from HNSCC patients treated with cetuximab-based therapy and analyzed for association with progression free survival (PFS).

**Results:**

Cetuximab induced pro-inflammatory cytokine secretion from HNSCC cells in vitro which was mediated by an IL-1α/IL-1R1/MyD88-dependent signaling pathway. IL-1 signaling blockade did not affect the anti-tumor efficacy of cetuximab, while increased IL-1α expression using polyanhydride nanoparticles in combination with cetuximab safely and effectively induced a T cell-dependent anti-tumor immune response. Detectable baseline serum levels of IL-1α were associated with a favorable PFS in cetuximab-based therapy-treated HNSCC patients compared to HNSCC patients with undetectable levels.

**Conclusions:**

Altogether, these results suggest that IL-1α in combination with cetuximab can induce a T cell-dependent anti-tumor immune response and may represent a novel immunotherapeutic strategy for EGFR-positive HNSCCs.

**Electronic supplementary material:**

The online version of this article (10.1186/s40425-019-0550-z) contains supplementary material, which is available to authorized users.

## Background

Although EGFR is highly expressed in HNSCC tumors, EGFR-targeted therapy using tyrosine kinase inhibitors (TKIs) has failed in clinical trials for HNSCC [1–4]. The EGFR monoclonal IgG_1_ antibody cetuximab is the only FDA approved EGFR inhibitor for first-line treatment of R/M HNSCC. Cetuximab-based therapy has relatively high response (36%) and disease control rates (81%) but is generally not curative and most patients will experience disease progression [5, 6]. Immunotherapy with antibodies targeting programmed cell death protein-1 (PD-1) have shown great promise and are now FDA approved for R/M HNSCC as second-line therapy. Single agent immunotherapy has only modest response rates (13–16%) yet these responses, in contrast to cetuximab-based therapy, are remarkably durable [7, 8]. However only a minority of patients derive benefit from single-agent immunotherapies and improvements are needed before routine use of anti-PD-1 agents as first-line treatment for R/M disease. Nevertheless, the relatively high response rates of cetuximab-based therapy and the durable responses of immunotherapy provide a strong rationale for the development of novel EGFR-targeted/immunotherapy combination regimens.

Previous work in our laboratory has shown that EGFR inhibitors induce an upregulation of a variety of inflammatory and immune response pathways via IL-1 alpha (IL-1α) release [9, 10]. The IL-1 pathway plays a critical role in the regulation of immune and inflammatory responses to infections and sterile insults [11, 12]; and dysregulation of this pathway is involved in a number of autoinflammatory disorders (e.g. fever, rashes, arthritis, and organ-specific inflammation) [11]. The IL-1 pathway is triggered when the ligands IL-1α and IL-1 beta (IL-1β) bind to IL-1 receptor type I (IL-1R1) that forms a complex with the IL-1 receptor accessory protein (IL-1RAcP) and then recruits myeloid differentiation primary response gene 88 (MyD88), IL-1 receptor-associated kinases (IRAKs), and TRAF6 [11]. This pathway results in NFκB and MAPK signaling leading to the expression of IL-1 target genes that are involved in inflammation and immune responses including additional IL-1 ligands that promote a positive feed forward loop [11].

The clinical relevance of IL-1 pathway activation in the context of tumor response to cetuximab is unclear. IL-1 ligand gene expression has been associated with poor prognosis and can induce the expression of a variety of pro-tumor survival factors involved in immune cell recruitment and angiogenesis [13, 14]. In fact, our previous work has shown that IL-1 signaling plays an important role in resistance to the EGFR TKI erlotinib in HNSCC cells [15]. Conversely, IL-1 signaling has been shown to play a role in tumor suppression via natural killer (NK) and T cell-mediated cytotoxicity [16–22] which are also major anti-tumor mechanisms of cetuximab [23–26]. Based on the overlapping roles of both cetuximab and IL-1 signaling in anti-tumor immune responses, we investigated if increasing IL-1 signaling may represent an effective immunotherapeutic strategy to combine with cetuximab. We additionally explore the potential of circulating IL-1α levels as a predictive biomarker of response to cetuximab-based chemotherapy in a limited cohort of R/M HNSCC patients.

## Materials and methods

### Cell lines

Cal-27 HNSCC cells were obtained from the American Type Culture Collection (ATCC, Manassas, VA, USA). SQ20B cells were gifted to our lab from Dr. Anjali Gupta (Department of Radiation Oncology, University of Iowa, IA, USA). MyD88 and IL-1R1 knockdown SQ20B cells were generated as previously described here [9]. IL-1α overexpressing SQ20B cells were generated using Myc-DDK-tagged-human IL-1α cDNA (Origene). TUBO-EGFR [27, 28] cells were gifted to our lab from Dr. Yang-Xin Fu (Department of Pathology, University of Chicago, Chicago, IL, USA). All cell lines were passaged no more than 15 times. All HNSCC cell lines are EGFR positive and are sensitive to EGFR inhibitors. Cal-27 and SQ20B cells were cultured in Dulbecco’s Modified Eagle’s Medium (DMEM) supplemented with 10% fetal bovine serum (FBS) and 0.1% gentamycin. TUBO-EGFR cells were cultured in DMEM supplemented with 10% FBS, 10 mM HEPES, 1% non-essential amino acids, and 1% penicillin/streptomycin. All cells are adherent and were cultured in vented flasks at 37 °C and 5% CO_2_ in a humidified incubator.

### In vitro drug treatment

Cetuximab and anakinra (ANA) were purchased from the inpatient pharmacy at the University of Iowa Hospitals and Clinics. Human IgG was purchased from Sigma-Aldrich (St. Louis, MO). Recombinant human IL-1α (rIL-1α) and IL-1β (rIL-1β) were purchased from R & D Systems (Minneapolis, MN). Neutralizing human and mouse IL-1α (nIL-1α) and IL-1β (nIL-1β) antibodies were purchased from Invivogen (San Diego, CA). Drugs were added to cells at final concentrations of 1–100 μg/mL cetuximab, 10 μg/mL ANA, 100 μg/mL IgG_1_, 1 μg/mL nIL-1α/nIL-1β and 0.5 ng/mL rIL-1α/rIL-1β. The required volume of each drug was added directly to complete cell culture media on cells to achieve the indicated final concentrations for up to 48 h.

### IL-1α polyanhydride nanoparticle synthesis

IL-1α-loaded polyanhydride nanoparticles were synthesized using the anhydride monomers 1,8-bis(*p*-carboxyphenoxy)-3,6-dioxaoctane (CPTEG) and 1,6-bis(*p*-carboxyphenoxy) hexane (CPH). First, a 20:80 CPTEG:CPH copolymer was synthesized via melt polycondensation as previously described [29, 30]. Next, 20:80 CPTEG:CPH nanoparticles encapsulating murine rIL-1α (Biolegend, San Diego, CA) were synthesized using a solid-oil-oil double emulsion process [31]. Briefly, 20:80 CPTEG:CPH polymer containing 1.5 wt.% rIL-1α was dissolved 20 mg/mL in methylene chloride. The solution was sonicated for 30 s to ensure the components were fully dissolved and evenly distributed. Nanoparticles were then precipitated into chilled pentane (1:250 methylene chloride:pentane) and collected via vacuum filtration. Nanoparticle morphology was verified with scanning electron microscopy (FEI Quanta 250, FEI, Hillsboro, OR) and their size analyzed with ImageJ (ImageJ 1.48v, NIH). To observe the release kinetics of IL-1α, nanoparticle suspensions of 10 mg/mL in PBS were sonicated to disperse particle aggregates and incubated at 37 °C for approximately one week. Periodically, the suspensions were centrifuged, the supernatant was collected, and the particles resuspended in fresh PBS. The amount of released IL-1α in the supernatant was measured using a microBCA assay (Thermo Fisher Scientific, Waltham, MA). At the end of the experiment, the remaining nanoparticles were placed in 40 mM sodium hydroxide to extract any remaining protein. The encapsulation efficiency was determined by comparing the total amount of protein released and extracted from the nanoparticles to the amount theoretically encapsulated.

### Enzyme-linked immunosorbent assay

Levels of IL-6, IL-8, IL-1α and IL-1β in the cell culture media of drug-treated cells and levels of IL-1α in human serum were determined by ELISA. Each cytokine was detected according to the manufacturer’s protocol using Human Quantikine ELISA Kits (R&D Systems, Minneapolis, MN). Colorimetric analysis was performed using a Synergy H1 Hybrid Multi-Mode Reader (BioTek, Winooski, VT).

### Western blot analysis

Cell lysates were standardized for protein content, resolved on 4–12% SDS polyacrylamide gels, and blotted onto nitrocellulose membranes. Membranes were probed with rabbit anti-MyD88 (1:500, Cell Signaling), anti-IL-1R1 (1:500, Santa Cruz), and anti-beta-actin (1:5000, Thermo Scientific). Antibody binding was detected by using an ECL Chemiluminescence Kit (Amersham).

### Tumor cell implantation

Male and female athymic *nu/nu* or BALB/c mice (4–6 weeks old) were purchased from Envigo Laboratories (Huntingdon, Cambridgeshire, United Kingdom). Mice were housed in a pathogen-free barrier room in the Animal Care Facility at the University of Iowa and handled using aseptic procedures. All procedures were approved by the IACUC committee of the University of Iowa and conformed to the guidelines established by the NIH. Mice were allowed at least 3 days to acclimate prior to beginning experimentation, and food and water were made freely available. SQ20B or Cal-27 cells (1 × 10^6^ cells/mouse) were inoculated into athymic nude mice and TUBO-EGFR cells (5 × 10^5^ cells/mouse) were inoculated into BALB/c mice by subcutaneous injection of 0.1 mL aliquots of saline containing cancer cells into the right flank using 26 gauge needles.

### In vivo drug administration

Drug treatment commenced 3 days after tumor inoculation. For the IL-1 blockade experiments, male and female Cal-27 and SQ20B tumor-bearing athymic *nu/nu* mice (*n* = 12 mice/group, 6 male/6 female) and male and female TUBO-EGFR-bearing BALB/c mice (*n* = 10 mice/group, 5 male/5 female) were randomized into the following treatment groups: Control group - Mice were administered saline daily and 2 mg/kg IgG i.p twice per week. IL-1R antagonist (anakinra [ANA]) group - ANA was administered at 10 mg/kg i.p. daily. Cetuximab (CTX) group - CTX was administered at 2 mg/kg (or 8 mg/kg for TUBO-EGFR tumors [32]) i.p. twice per week. CTX + ANA group - mice were administered both CTX and ANA i.p. at the doses/schedules indicated above. For the IL-1α overexpressing experiment, female athymic *nu/nu* mice (*n* = 4–5 mice/group) bearing IL-1α overexpressing xenografts (#20) or control transfected xenografts (#16) were treated with either CTX (2 mg/mouse twice/week i.p.) or IgG as a control. For the IL-1 pathway activation experiments, male and female athymic *nu/nu* mice (*n* = 10 mice/group, 5 male/5 female) bearing SQ20B xenograft tumors or BALB/c mice (n = 10 mice/group, 5 male/5 female) bearing TUBO-EGFR tumors were treated with 2 mg/kg (or 8 mg/kg for TUBO-EGFR tumors) CTX i.p. twice/week with or without 0.6 μg human rIL-1α (for SQ20B tumors) or murine rIL-1α (for TUBO-EGFR tumors). IgG and H_2_O were used as controls. IL-1α was given at least half an hour prior to CTX or IgG administration, and again 24 h later totaling 4 doses of CTX and IgG and 8 doses of IL-1α and H_2_O. For the IL-1α nanoparticle experiment, female BALB/c mice (*n* = 9–10 mice/group) bearing TUBO-EGFR tumors were treated with 8 mg/kg CTX i.p. twice/week with or without IL-1α-NPs (0.5 mg NPs containing 7.5 μg IL-1α, on the first day of treatment). IgG and empty nanoparticles (EMP-NP) were used as controls. For the T cell depletion experiments, female BALB/c mice (n = 9–10 mice/group) bearing TUBO-EGFR tumors were treated with cetuximab (CTX, 8 mg/kg twice/week) in combination with a single i.p. dose on treatment day 1 of IL-1α-NPs with or without anti-CD4 (100 μg (clone GK1.5)) or anti-CD8 (300 μg (clone 53–6.7)) 1 and 3 days prior to tumor inoculation, and every 3–4 days after tumor inoculation. All treatments were given for the duration of 2 weeks with exception of the IL-1α overexpressing SQ20B experiment where treatment ended after 3 weeks. Mice were evaluated daily and tumor measurements and weights were taken three times per week using Vernier calipers. Tumor volumes were calculated using the formula: tumor volume = (length × width^2^)/2 where the length was the longest dimension, and width was the dimension perpendicular to length. Mice were euthanized via CO_2_ gas asphyxiation when tumor diameter exceeded 1.5 cm in any dimension. Tumor growth curves were plotted over time and stopped after a mouse in any treatment group reached euthanasia criteria.

### Flow cytometry

For the assessment of splenocytes and tumor-infiltrating immune cells, spleens and tumors were harvested immediately after sacrificing mice via CO_2_ asphyxiation. Tumors were digested with 100 U/mL collagenase type I and 100 μg/mL DNAse in RPMI + 10% FBS at 37 °C for 30 min. Digested tumors and spleens were forced through 70 μm filters and washed 3 times with FACS separation buffer supplemented with 10% FBS. Cells were washed with FACS buffer without FBS, counted, pelleted, and resuspended at 1 × 10^6^ cells / 100 μL. Cells were stained with various murine antibodies including CD3 (GK1.5 and 17A2), CD4 (GK1.5), CD8 (53–6.7), CD19 (6D5), CD25 (PC61), CD49b (DX5), CD69 (H1.2F3), CD122 (TM-β1), KLRG1 (2F1/KLRG1) and PD-1 (29F.1A12) (BioLegend) for 30 min at 4 °C protected from light. After staining, cells were washed with FACS buffer, and resuspended in 2% paraformaldehyde in FACS buffer (50 μL/well). Flow cytometry was performed using a BD FACSCANTO II cytometer.

### HNSCC patient study

Baseline serum samples from 11 recurrent and/or metastatic (R/M) HNSCC patients (Additional file 1: Table S1) scheduled for cetuximab-based chemotherapy (i.e. carboplatin, cisplatin, 5-fluorouricil [5-FU], paclitaxel) at the University of Iowa Hospitals and Clinics (UIHC) Holden Comprehensive Cancer Center (HCCC) were collected. Serum IL-1α levels were measured by ELISA and interrogated for associations with clinical outcomes. This study was approved by the University of Iowa Institutional Review Board (IRB approval #201302782). This study was conducted in accordance with ethical standards presented in the 2013 Declaration of Helsinki. All subjects provided their informed consent in written form for participation in the study.

### Statistical analysis

Statistical analysis was done using GraphPad Prism version 5 for Windows (GraphPad Software, San Diego, CA). Differences between 3 or more means were determined by one-way ANOVA with Tukey post-tests. Two-way ANOVA will be used to determine differences among cell lines and drug treatment groups. Linear mixed effects regression models were used to estimate and compare the group-specific change in tumor growth curves. Kaplan-Meier survival curves were generated to illustrate the different survival rates over time. Differences in median survival were determined by Log-rank (Mantel-Cox) test. All statistical analysis was performed at the *p* < 0.05 level of significance.

## Results

### Cetuximab induces secretion of pro-inflammatory cytokines

**Fig. 1:**
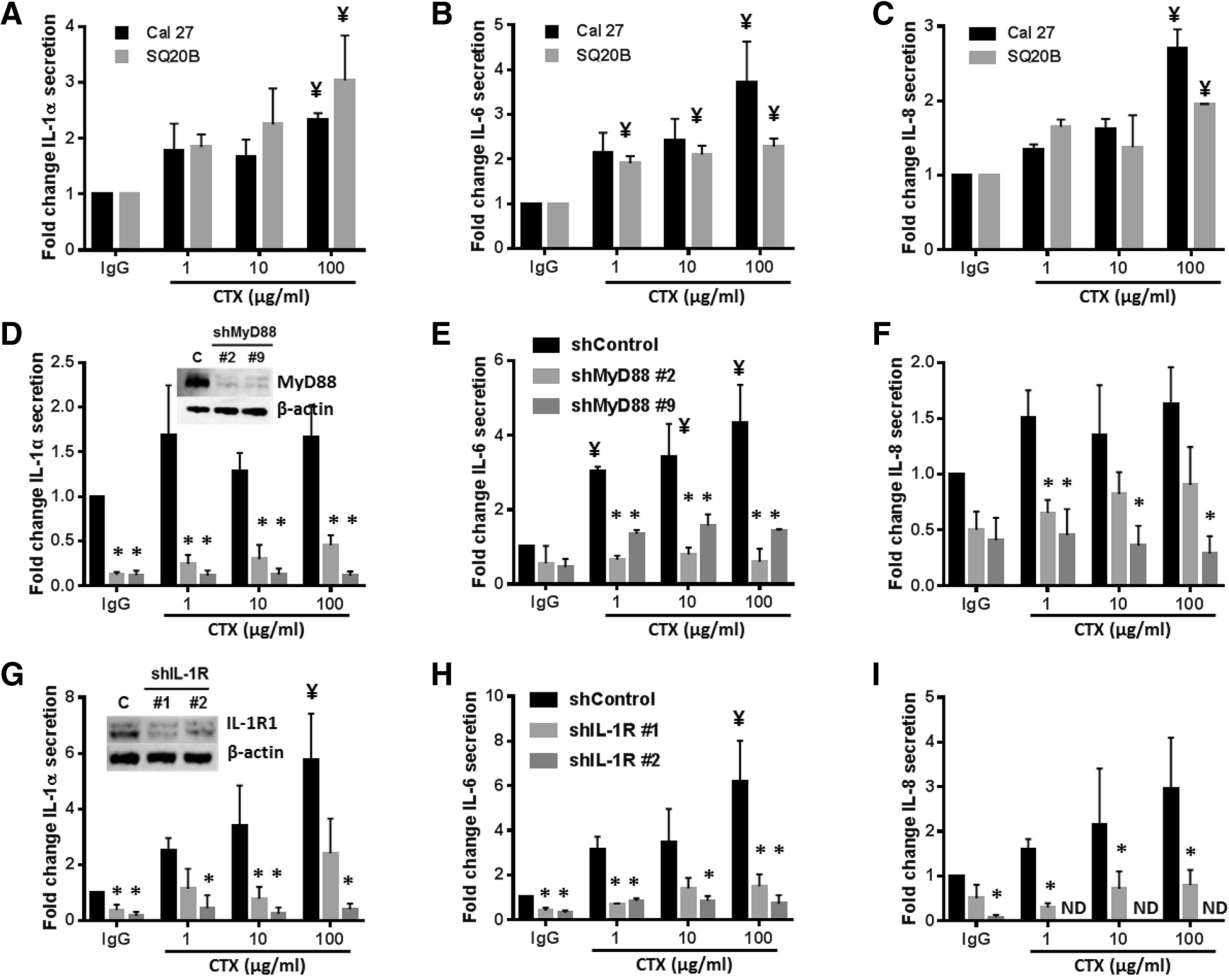
Cetuximab induces secretion of pro-inflammatory cytokines via IL-1R/MyD88 signaling. Cal 27 and SQ20B HNSCC cells (**a**-**c**), SQ20B cells derived from MyD88 stable knockout clones (shMyD88 #2, shMyD88 #9) and control cells (shControl) (**d**-**f**), and SQ20B cells derived from IL-1R1 stable knockout clones (shIL-1R #1, shIL-1R #2) and control cells (shControl) (**g**-**i**) were treated with 1–100 μg/mL cetuximab (CTX) or 100 μg/mL IgG for 48 H*. media* was collected and ELISAs were performed to measure IL-1α (**a**, **d**, **g**), IL-6 (**b**,**e**,**h**), and IL-8 (**c**,**f**,**i**). Cells were analyzed for expression of MyD88 (D inset) and IL-1R1 (G inset) by Western blot and β-actin was used as a control. Error bars = SEM. *N* = 3. ^¥^:*p* < 0.05 vs. respective IgG treatment; *p < 0.05 vs. respective shControl cell line. ND = not detected

To determine if cetuximab triggers proinflammatory cytokine release from HNSCC cells, Cal-27 and SQ20B cells were treated with 1, 10 and 100 μg/mL cetuximab for 48 h and cell culture media was analyzed for IL-1α, IL-6 and IL-8 by ELISA. In Cal-27 cells cetuximab at a dose of 100 μg/mL significantly increased IL-1α (Fig. 1A), IL-6 (Fig. 1B) and IL-8 (Fig. 1C), while in SQ20B cells cetuximab increased IL-1α (Fig. 1A) and IL-8 (Fig. 1C) at 100 μg/mL and IL-6 at all cetuximab doses tested (Fig. 1B). The observed effects did not appear to be dose-dependent in general. IL-1β was not detectable in any of the treated samples (data not shown). These data show that cetuximab has the ability to trigger the release of pro-inflammatory cytokines directly from HNSCC cells.

### MyD88 knockdown suppresses cetuximab-induced cytokine secretion

A well-established mechanism of pro-inflammatory cytokine production involves the cytosolic adaptor protein MyD88, which acts through intermediaries to induce NFκB activation and cytokine expression [33]. Cell lines derived from MyD88 stable knockout clones (shMyD88#2, shMyD88#9, Fig. 1D inset) demonstrated significantly reduced IL-1α (Fig. 1D), IL-6 (Fig. 1E) and IL-8 (Fig. 1F) in the presence of cetuximab compared to control cells (shControl) with the exception of IL-8 secretion from the shMyD88#2 clone at 10 and 100 μg/mL (Fig. 1F). MyD88 knockdown also significantly reduced IL-1α baseline levels (Fig. 1D). These data support the role of MyD88-dependent signaling in cetuximab-induced cytokine production.

### IL-1R1 knockdown suppresses cetuximab-induced cytokine secretion

**Fig. 2:**
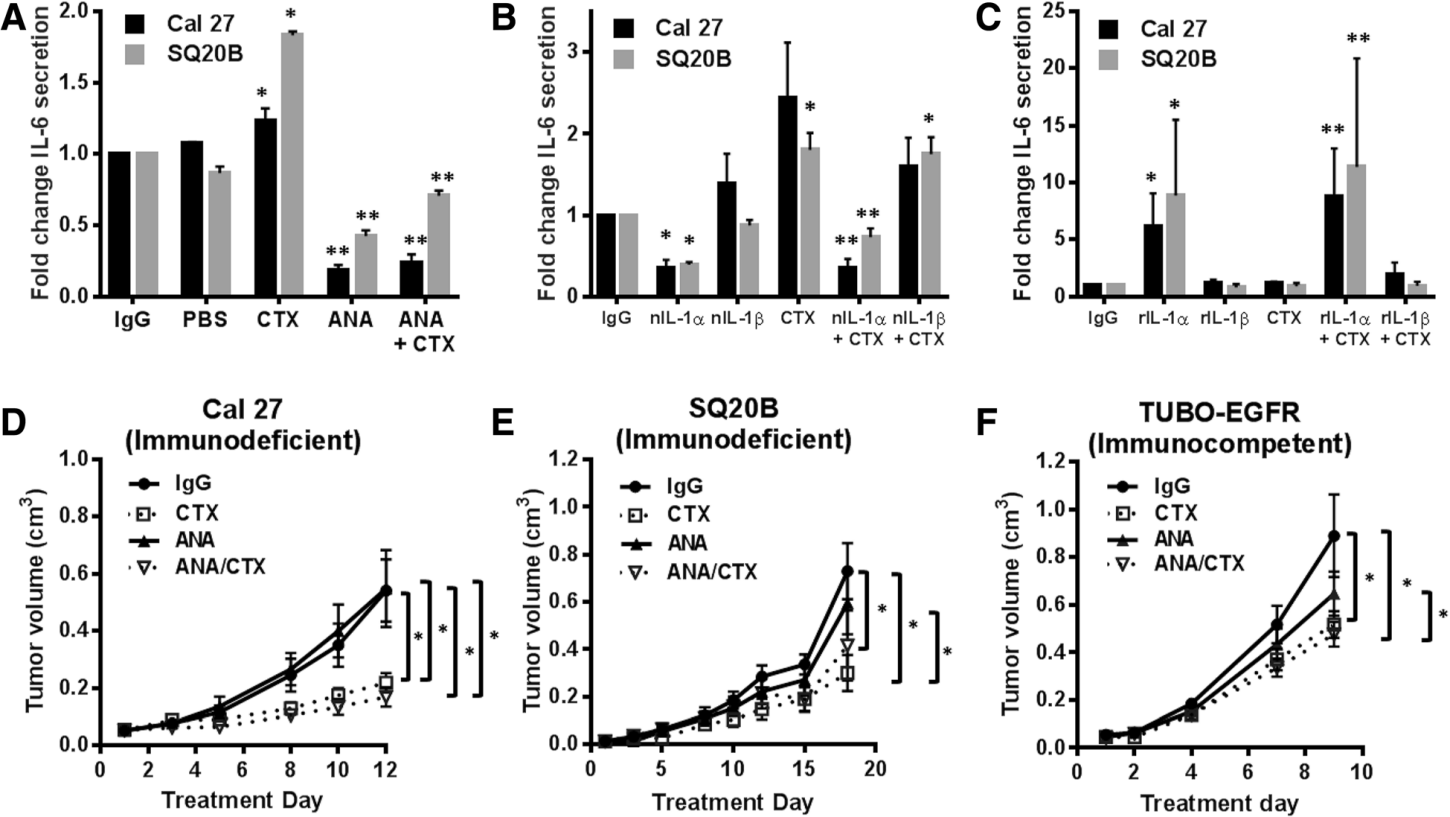
IL-1 blockade does not improve the anti-tumor efficacy of cetuximab. **a** Cal 27 and SQ20B HNSCC cells were pretreated with 10 μg/mL anakinra (ANA) for 4 h with or without 100 μg/mL cetuximab (CTX) for 48 h. IgG and PBS were used as controls. **b** Cal 27 and SQ20B HNSCC cells were pretreated with 1 μg/mL nIL-1αab or 1 μg/mL nIL-1βab for 4 h with or without 100 μg/mL CTX for 48 h. IgG was used as a control. **c** Cal 27 and SQ20B HNSCC cells were pretreated with 0.5 ng/mL human rIL-1α or 0.5 ng/mL human rIL-1β for 4 h with or without 100 μg/mL CTX for 48 h. IgG was used as a control. Media was collected and ELISAs were performed to measure IL-6 secretion. N = 3–4. *: *p* < 0.05 vs. IgG; **: *p* < 0.05 vs CTX and IgG. **d**-**f**: Athymic *nu/nu* mice (*n* = 12 [*n* = 6 male/n = 6 female]) bearing Cal 27 (**d**) and SQ20B (**e**) tumors and BALB/c mice (*n* = 10 [*n* = 5 male/n = 5 female]) bearing TUBO-EGFR tumors (**f**) were treated with CTX (2 mg/kg [8 mg/kg for TUBO-EGFR tumors]) twice weekly and anakinra (10 mg/kg daily) i.p. for two weeks. Tumors were measured three times/week. Tumor growth curves shown were stopped after a mouse in any treatment group reached euthanasia criteria. Error bars = SEM. *:*p* < 0.05

MyD88 is required for the activity of members of the Toll/Interleukin-1 receptor (TIR) superfamily which include Toll-like Receptors (TLRs), the IL-1R, and the IL-18 Receptor (IL-18R) [33]. Activation of these receptors lead to the recruitment of MyD88 via its TIR domain, resulting in NFκB activation and expression of pro-inflammatory cytokines including IL-1α, IL-6 and IL-8 [33]. We previously found that erlotinib-induced secretion of pro-inflammatory cytokines was mediated by IL-1R/MyD88-dependent signaling (and not TLR or IL-18R signaling [9]), therefore we determined if these results could be duplicated with cetuximab. Cell lines derived from IL-1R1 stable knockout clones (shIL-1R1#1, shIL-1R1#2, Fig. 1G inset) were assessed and demonstrated significantly reduced IL-1α at baseline and in the presence of cetuximab (with exception of the shIL-1R1#1 clone at 1 and 100 μg/mL (Fig. 1G)) supporting previous reports of IL-1α as a gene target of IL-1 signaling and the feed-forward nature of IL-1 signaling. The IL-1R1 knockout clones also demonstrated significantly reduced IL-6 at baseline and in the presence of cetuximab with exception of the shIL-1R1#1 clone at 10 μg/mL (Fig. 1H); and significantly reduced IL-8 in the presence of cetuximab in the shIL-1R1#1 clone at all doses tested (Fig. 1I). In the shIL-1R#2 clone, IL-8 was significantly suppressed at baseline and not detected in response to cetuximab at all doses tested (Fig. 1I). Furthermore, using IL-6 as an endpoint of IL-1 signaling, we showed that pretreatment with the IL-1 receptor antagonist (IL-RA/anakinra [ANA]) significantly reduced baseline and cetuximab-induced secretion of IL-6 from both cell lines (Fig. 2A). Together these results point to the induction of cytokine secretion via an IL-1R/MyD88-dependent pathway in response to cetuximab.

### Cetuximab-induced IL-1 signaling is triggered by IL-1α release

To confirm which ligand(s) (IL-1α or IL-1β) may be responsible for activating the IL-1 pathway, we again used IL-6 as an endpoint since IL-1 signaling is well known to trigger the release of IL-6 via IL-1R1 binding [34]. We found that neutralization of IL-1α (but not IL-1β) significantly suppressed cetuximab-induced IL-6 secretion (Fig. 2B) suggesting that IL-1α in particular may be responsible for cetuximab-induced IL-1R1/MyD88 signaling and cytokine (including IL-6) release. Exogenous human rIL-1α dramatically and significantly increased IL-6 secretion in the absence and presence of cetuximab (Fig. 2C) supporting IL-6 expression/secretion as an endpoint of IL-1 signaling. Surprisingly, this effect was not observed with rIL-1β even though both ligands bind the same receptor (Fig. 2C). Together these results suggest that IL-1α, but not IL-1β, is responsible for activation of IL-1R1/MyD88 signaling and cytokine secretion triggered by cetuximab in HNSCC cells.

### IL-1 blockade does not affect the anti-tumor activity of cetuximab

Given that IL-1 signaling may play both pro-survival and anti-tumor roles in cancer biology, we wanted to determine the clinical relevance (if any) of cetuximab-induced IL-1 signaling. We showed that IL-1 blockade using anakinra (which binds both human and mouse IL-1R1) did not significantly affect tumor response to cetuximab in Cal-27 (Fig. 2D) and SQ20B xenograft tumors (Fig. 2E). Similar results were observed in an immunocompetent TUBO-EGFR/BALB/c syngeneic mouse model (Fig. 2F) which utilizes the murine TUBO cell line [28] that was transfected with human EGFR [26, 32] - since cetuximab binds to human and not murine EGFR. Interestingly, we found that in the Cal-27 xenograft model, tumor-bearing female mice demonstrated significantly increased tumor growth in response to anakinra as a single agent (Additional file 2: Figure S1A), whereas male mice did not demonstrate this phenomenon (Additional file 2: Fig. S1B). These sex differences were not observed in the SQ2OB (Fig. 2E) or TUBO-EGFR (Fig. 2F) mouse models. Altogether the data suggests that IL-1 blockade did not significantly affect the anti-tumor efficacy of cetuximab.

### Increased IL-1α may enhance the anti-tumor efficacy of cetuximab in vivo

**Fig. 3:**
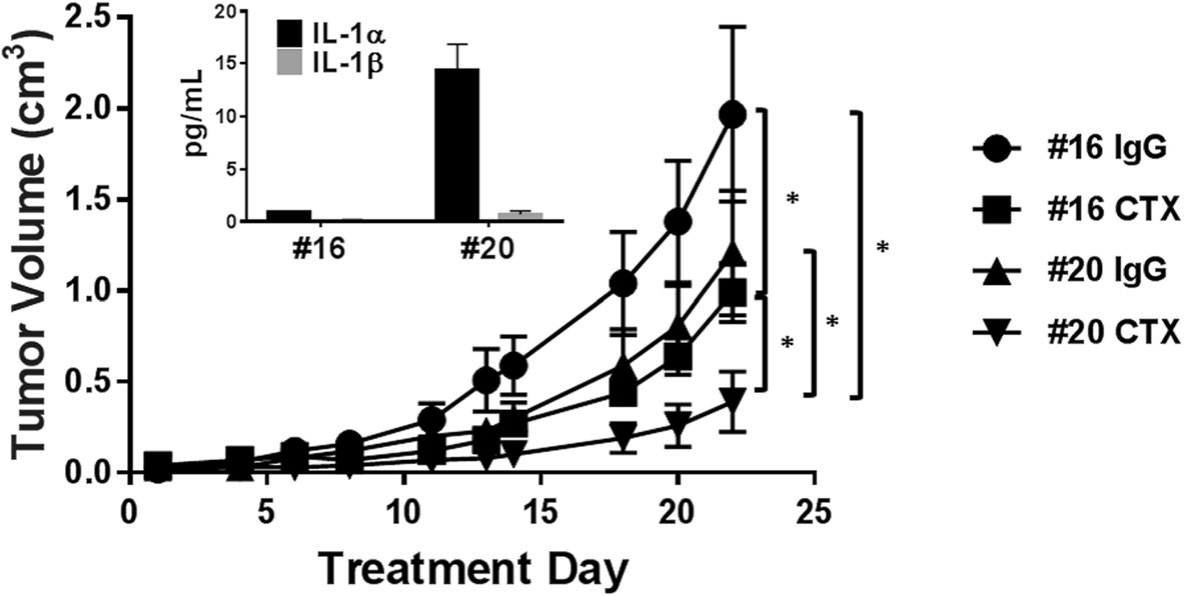
IL-1α overexpression enhances the anti-tumor efficacy of cetuximab. Female athymic *nu/nu* mice bearing IL-1α overexpressing (#20) or control (#16) SQ20B tumors were treated with cetuximab (CTX, 2 mg/kg, twice/week) or IgG for 3 weeks. Overexpression was confirmed by ELISA (inset). Tumors were measured three times weekly. Tumor growth curves shown were stopped after a mouse in any treatment group reached euthanasia criteria. Error bars = SEM. *N* = 4–5 mice/treatment group. *: *p* < 0.05

Since IL-1 signaling may be involved in anti-tumor response, we next sought to address if increasing IL-1α expression could enhance the efficacy of cetuximab. To accomplish this we overexpressed IL-1α in the SQ20B cell line using Myc-DDK-tagged-human IL-1α cDNA (Origene). An overexpressing IL-1α clone (#20, Fig. 3 inset) and 1 control clone (#16, Fig. 3 inset) were grown in a SQ20B xenograft model in female athymic nude mice in the presence of IgG or cetuximab. We found that the IL-1α-overexpressing tumors treated with cetuximab (#20 CTX) grew significantly slower compared to the IgG (#16 IgG) and cetuximab-treated (#16 CTX) control clones, and the IgG-treated IL-1α-overexpressing clones (#20 IgG) (Fig. 3) suggesting that increased tumor-derived IL-1α may enhance tumor response to cetuximab.

### Systemic delivery of recombinant IL-1α demonstrates anti-tumor activity in immunocompetent mice

**Fig. 4:**
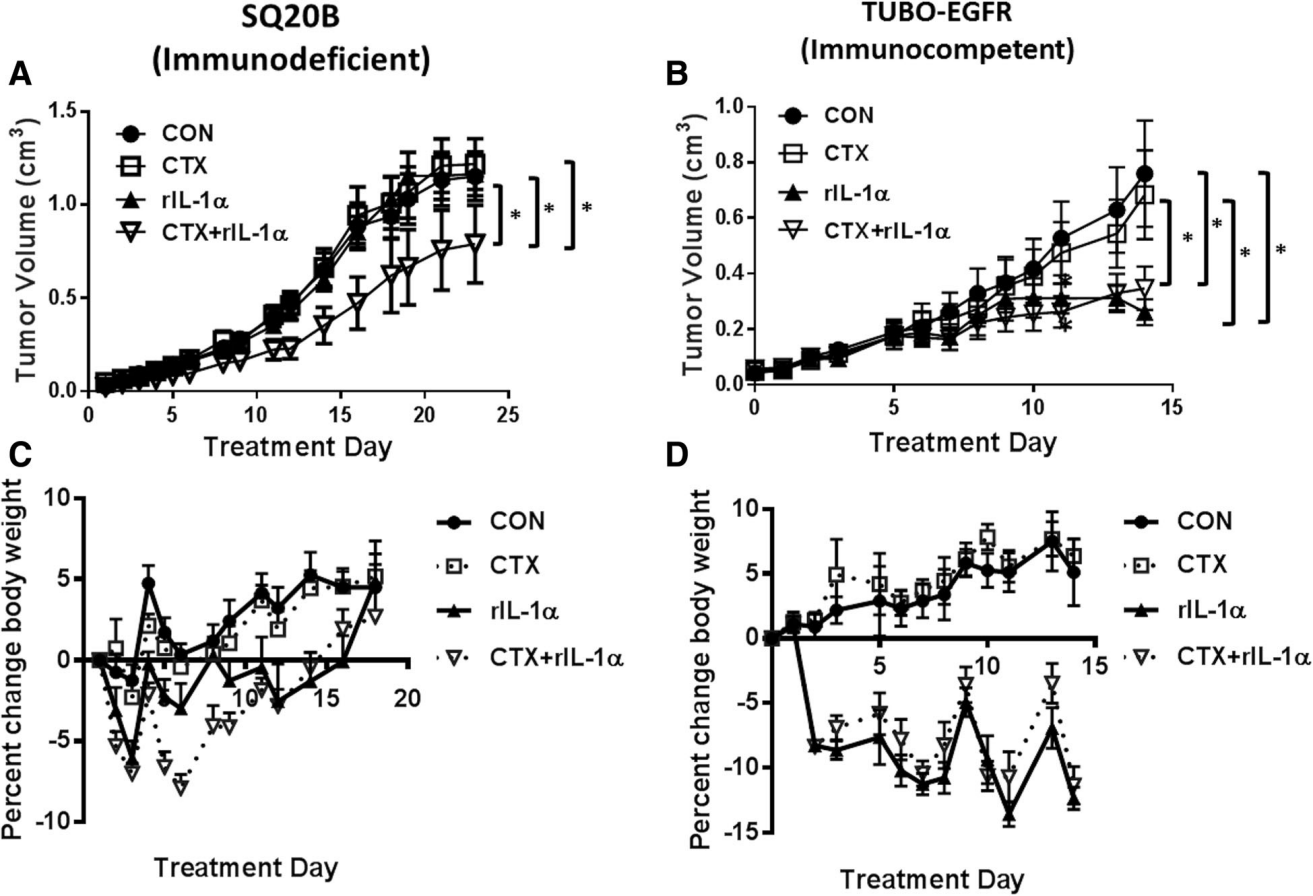
Tumor response to recombinant IL-1α differs between immunodeficient and immunocompetent mouse models. Athymic *nu/nu* mice (n = 10 [n = 5 male/n = 5 female]) bearing SQ20B tumors (**a**, **c**) or BALB/c mice (n = 10 [n = 5 male/n = 5 female]) bearing TUBO-EGFR tumors (**b**, **d**) were treated with cetuximab (CTX, 2 mg/kg [8 mg/kg for TUBO-EGFR tumors], twice/week) with or without 0.6 μg human (**a**, **c**) or murine (**b**, **d**) recombinant IL-1α (rIL-1α) for 2 weeks. IgG and H_2_O were used as controls. IL-α was given at least half an hour prior to CTX or IgG administration, and again 24 h later totaling 4 doses of CTX and IgG, and 8 doses of IL-1α and H_2_O. Tumor growth (**a**, **b**) and mouse weights (C,D) were measured 3–5 times per week. Tumor growth curves shown were stopped after a mouse in any treatment group reached euthanasia criteria. Error bars = SEM. *: *p* < 0.05

We next investigated if systemic i.p. delivery of recombinant IL-1α (rIL-1α) would enhance tumor response to cetuximab. In SQ20B tumor-bearing athymic nude mice we observed that cetuximab nor human rIL-1α showed significant anti-tumor activity during the 2 week treatment period (Fig. 4A) however, tumor growth in the human rIL-1α in combination with cetuximab-treatment group was significantly slower compared to the other treatment groups (Fig. 4A). Note that human rIL-1α can bind to murine IL-1R1 [35]. When this experiment was attempted in the immunocompetent TUBO-EGFR/BALB/c syngeneic mouse model [26, 28, 32] using murine rIL-1α, we again observed no significant difference with cetuximab as a single agent compared to control-treated tumors (Fig. 4B) over the 2 week treatment period. The non-significance of cetuximab in this tumor model was driven by the female mice (Additional file 3: Figure S2B) which did not respond to cetuximab in contrast to the male mice which did respond (Additional file 3: Figure S2A). Murine rIL-1α as a single agent was equally as effective as rIL-1α + cetuximab at significantly suppressing tumor growth (Fig. 4B), although this observation may again be influenced by gender differences since murine rIL-1α as a single agent demonstrated superior anti-tumor activity compared to all other treatment groups in female mice (Additional file 3: Figure S2B) and not male mice (Additional file 3: Figure S2A). We summarize here that in immunocompetent mice, IL-1α as a single agent and in combination with cetuximab demonstrates anti-tumor activity and that gender differences influence drug response in this mouse model.

### Treatment with recombinant IL-1α triggers weight loss

Although these data demonstrate the anti-tumor properties of rIL-1α in immunodeficient mice in combination with cetuximab (Fig. 4A) and as a single agent in immunocompetent mice (Fig. 4B), unfortunately (but not unexpectedly) all mice that were treated with human or mouse rIL-1α in these experiments exhibited significant side effects including weight loss (Fig. 4C, D), diarrhea and lethargy. These side effects resulted in shortening the drug treatment time from 3 weeks to 2 weeks as originally planned. In the immunodeficient mice, there was an initial decrease in body weight in all mice treated with human rIL-1α over the first week of treatment which gradually recovered to that of non-rIL-1α-treated mice (Fig. 4C) after the 2 week treatment period. In the immunocompetent model, the body weights of murine rIL-1α-treated BALB/c mice steadily declined over the 2 week treatment period (Fig. 4D) with no recovery as observed in the immunodeficient mice (Fig. 4C) resulting in the euthanization of these mice and early termination of the experiment. Intestines (including small and large intestine with attached mesenteric fat and sometimes pancreas) were made into Swiss rolls and evaluated to determine, if possible, the cause of diarrhea in these mice. There was multifocal moderate and in some cases marked mesenteric inflammation, composed primarily of neutrophils, macrophages, lymphocytes and plasma cells in mice treated with rIL-1α (rIL-1α alone or CTX+ rIL-1α) while those in the non-rIL-1α-treatment groups (CON or CTX) did not show the same infiltrates (Additional file 4: Figure S3). These results suggest that the repeated i.p. administration of rIL-1α may be triggering a dramatic pro-inflammatory response resulting in peritonitis, diarrhea, and weight loss, and that alternate delivery methods of rIL-1α may be a promising strategy as a single agent and in combination with cetuximab.

### Nanoparticle delivery of IL-1α enhances cetuximab activity

**Fig. 5:**
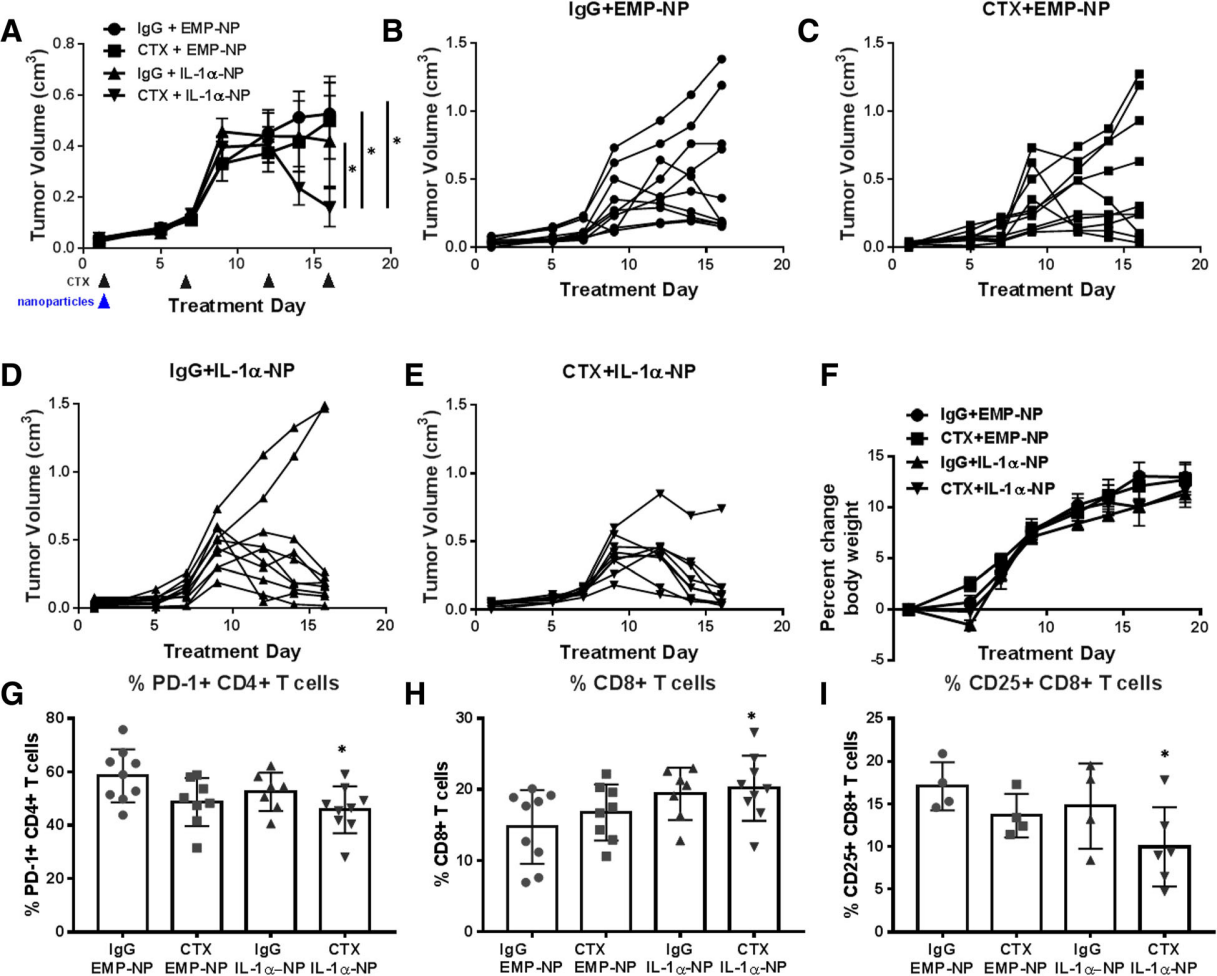
Nanoparticle delivery of IL-1α demonstrates anti-tumor efficacy. **a** Female BALB/c mice (*n* = 9–10 mice/treatment group) bearing TUBO-EGFR tumors were treated with cetuximab (CTX, 8 mg/kg, twice/week) for 2 weeks with or without a single administration of IL-1α nanoparticles (IL-1α-NPs (0.5 mg NPs containing 7.5 μg IL-1α)) on the first day of treatment. IgG and empty nanoparticles (EMP-NP) were used as controls. Tumor volumes were measured three times per week. Tumor growth curves shown were stopped after a mouse in any treatment group reached euthanasia criteria. *:*p* < 0.05. Error bars = SEM. **b**-**e**: Shown are spaghetti plots for each individual mouse in each treatment group shown in A. F: Mouse weights were measured three times per week. **g**-**i**: Spleens were harvested after therapy and PD-1 + CD4+ T cells (**g**), CD8+ T cells (**h**) and CD25 + CD8+ T cells (**i**) were analyzed using flow cytometry. Error bars = SDM. N = 4–9 per group. *: *p* < 0.05 vs IgG

In an attempt to circumvent the side effects observed with repeated i.p. rIL-1α delivery, we synthesized a polyanhydride nanoparticle formulation as a possible safer alternative method that would allow for prolonged IL-1α exposure with only a single administration to mice. A 20:80 CPTEG:CPH copolymer was used to encapsulate murine rIL-1α as a 1.5% IL-1α 20:80 CPTEG:CPH nanoparticle (IL-1α-NP) formulation. The IL-1α-NPs exhibited similar morphology and size (192.7 ± 67.5 nm; Additional file 5: Figure S4A) as seen in previous work [31]. The release kinetics of IL-1α demonstrated a burst release with greater than 90% of the payload being released within the first 24 h, and the remainder slowly released over the next 5 days (Additional file 5: Figure S4B). The encapsulation efficiency was found to be 61.6 ± 6.8%. Empty 20:80 CPTEG:CPH nanoparticles (EMP-NPs) were synthesized for use as a control. We observed that a single dose of IL-1α-NPs (0.5 mg NPs containing 7.5 μg IL-1α) i.p. on Day 1 of treatment in combination with cetuximab (8 mg/mouse, twice/week for 2 wks) (CTX + IL-1α-NP) to female TUBO-EGFR-bearing BALB/c mice demonstrated significantly reduced tumor growth compared to IgG + EMP-NP, CTX + EMP-NP, and IgG + IL-1α-NP treatment groups (Fig. 5A). However, when we plotted the tumor growth trajectory of individual mice from each treatment group (Fig. 5B-E), we observed that CTX + IL-1α-NP-treated mice caused complete tumor regression in almost all mice (8/9) in this treatment group (Fig. 5E) compared to IgG + EMP-NP (2/10, Fig. 5B), CTX + EMP-NP (3/10, Fig. 5C), and IgG + IL-1α-NP (6/10) (Fig. 5D). Importantly, there was no notable weight loss (Fig. 5F), diarrhea or other obvious side effects due to IL-1α-NP treatment over the treatment period. These data suggest that a single administration of a polyanhydride CPTEG:CPH nanoparticle formulation of IL-1α may be a relatively safe and effective option for IL-1α delivery as a single agent and in combination with cetuximab.

### Anti-tumor response to CTX + IL-1α-NP is T-cell dependent

**Fig. 6:**
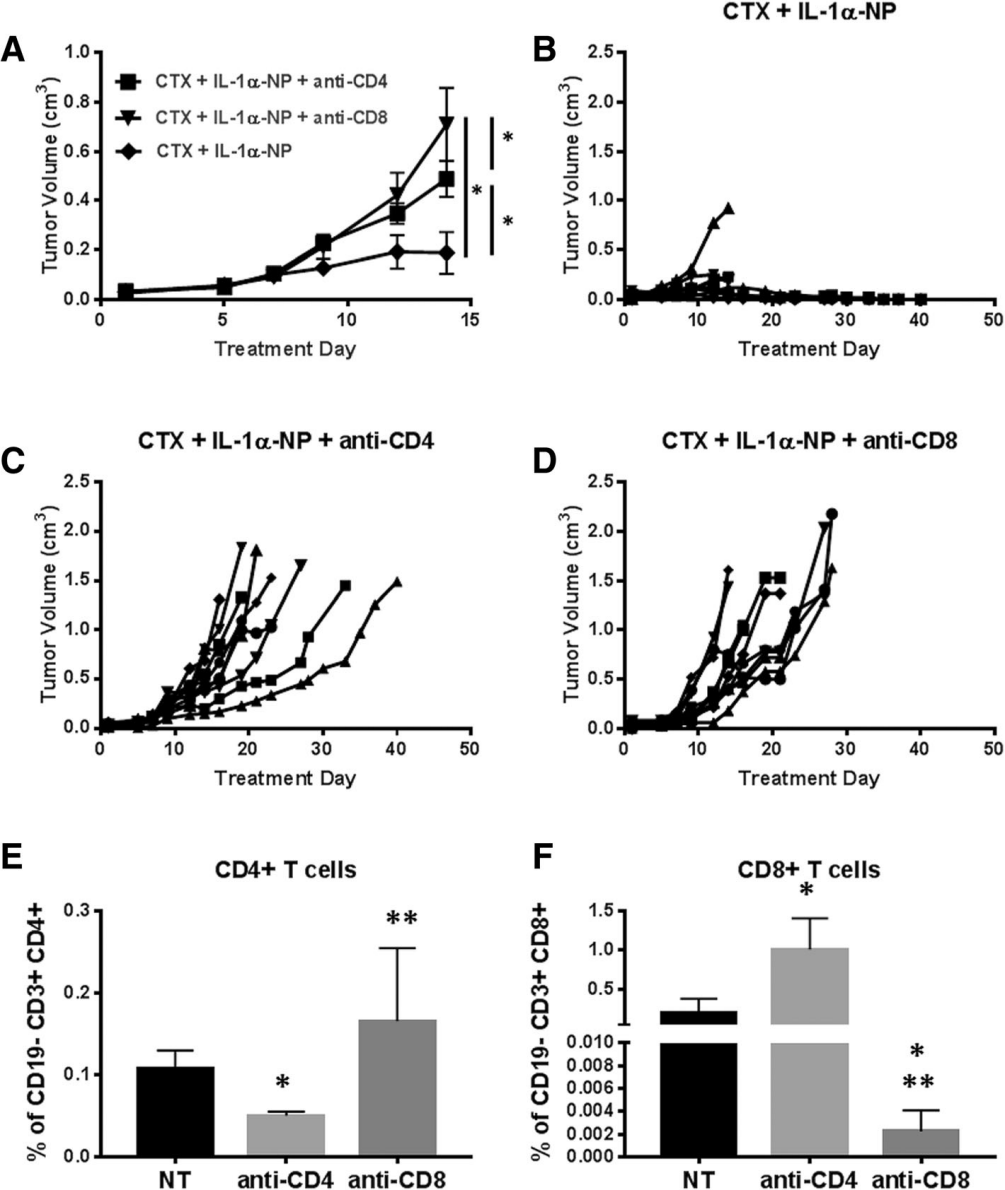
The anti-tumor effects of cetuximab+IL-1α-NP are T cell dependent. Female BALB/c mice (n = 9–10 mice/treatment group) bearing TUBO-EGFR tumors were treated with cetuximab (CTX, 8 mg/kg twice/week) in combination with a single i.p. dose on treatment day 1 of IL-1α-NPs (0.5 mg NPs containing 7.5 μg IL-1α) (CTX + IL-1α-NP (**a**,**b**) with or without anti-CD4 (100 μg (clone GK1.5)) (**a**,**c**) or anti-CD8 (300 μg (clone 53–6.7)) (**a**,**d**) 1 and 3 days prior to tumor inoculation, and every 3–4 days after tumor inoculation. Treatment duration was 3 weeks. Tumor volumes were measured three times per week. Tumor growth curves shown were stopped after a mouse in any treatment group reached euthanasia criteria. Error bars = SEM. *:*p* < 0.05. **b**-**d**: Shown are spaghetti plots for each individual mouse in each treatment group shown in **a**. Tumors from female BALB/c mice bearing TUBO-EGFR tumors (*n* = 3–4) were treated as described in A and harvested after 2 weeks of therapy for validation of CD4^+^ T cell (E) and CD8^+^ T cell (F) depletion by flow cytometry. *:*p* < 0.05 vs NT, **:*p* < 0.05 vs anti-CD4. Error bars = SDM

Because cetuximab in combination with rIL-1α showed some anti-tumor activity in athymic nude mice where NK cells (and not T cells) are present (Fig. 4A), and cetuximab along with IL-1 ligands have been previously reported to activate NK cell activity, we initially proposed that NK cells may be involved in the anti-tumor immune response to CTX + IL-1α-NP. However, immune cells isolated from spleens of CTX + IL-1α-NP-treated mice demonstrated no differences in the frequency of NK cells or activated NK cells in the spleen (Additional file 6: Figure S5) or tumors (Additional file 7: Figure S6) compared to the other treatment groups. However, spleens from mice administered CTX + IL-1α-NP showed significantly decreased percentages of PD-1^+^ CD4^+^ T cells (Fig. 5G), significantly increased percentages of CD8^+^ T cells (Fig. 5H), and significantly decreased CD25^+^ CD8^+^ T cells compared to IgG + EMP-NP (Fig. 5I). Percentages of CD69^+^ CD4^+^ and IFNγ^+^ CD8^+^ T cells were elevated in tumors from CTX + IL-1α-NP-treated mice but did not reach statistical significance compared to the other treatment groups (Additional file 8: Figure S7). To further interrogate the role of T cell-dependent immune response, female BALB/c mice bearing TUBO-EGFR tumors were treated with CTX + IL-1α-NP (Fig. 6A,B) as already described in Fig. 5 with or without anti-CD4 (100 μg (clone GK1.5)) (Fig. 6A,C) or anti-CD8 (300 μg (clone 53–6.7)) (Fig. 6A,D) 1 and 3 days prior to tumor inoculation, and every 3–4 days after tumor inoculation. Specific depletion of CD4+ (Fig. 6E) and CD8+ T cells (Fig. 6F) from tumors was confirmed by flow cytometry. Both CD4^+^ and CD8^+^ T cell depletion significantly reversed the anti-tumor effect of CTX + IL-1α-NP, and CD8^+^ T cell depletion was significantly more effective than CD4^+^ T cell depletion at reversing the anti-tumor effect of CTX + IL-1α-NP (Fig. 6A). Additionally spaghetti plots of individual mice in each of the treatment groups showed complete regression in 9/10 mice in the CTX + IL-1α-NP treatment group (Fig. 6B) compared to no tumor regression in the CTX + IL-1α-NP + anti-CD4 (Fig. 6C) and CTX + IL-1α-NP + anti-CD8 (Fig. 6D) treatment groups. These results suggest that the anti-tumor mechanism of CTX + IL-1α-NP involves a T cell-dependent immune response.

### Increased serum IL-1α levels may predict favorable progression-free survival (PFS) in R/M HNSCC patients treated with cetuximab-based therapy

**Fig. 7:**
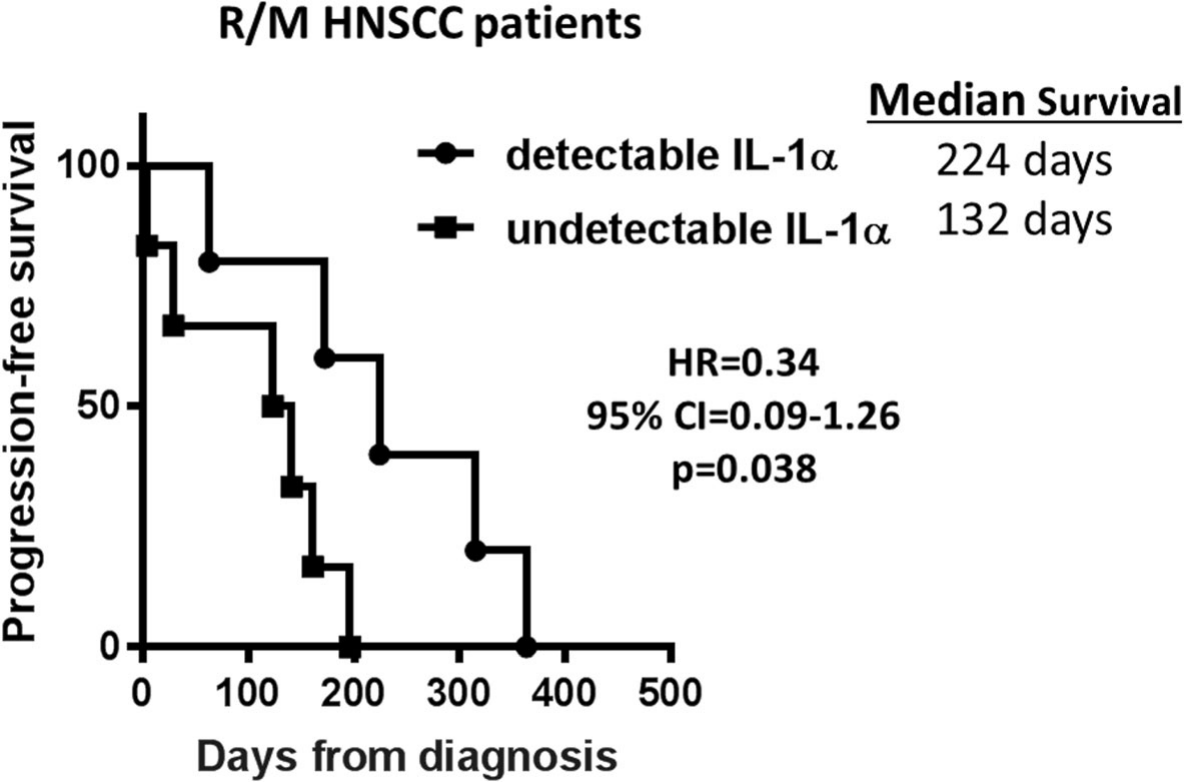
High serum IL-1α predicts progression free survival (PFS) in HNSCC patients treated with cetuximab-containing therapy. Baseline serum samples from 11 recurrent and/or metastatic (R/M) HNSCC patients scheduled for cetuximab-based chemotherapy (i.e. carboplatin, cisplatin, 5-FU, paclitaxel) at the University of Iowa Hospitals and Clinics Holden Comprehensive Cancer Center were collected. Serum IL-1α levels were measured by ELISA and patients were divided into two groups: detectable (n = 5) and undetectable (n = 6) IL-1α levels. Kaplan Meier survival curves were plotted for PFS for both groups. HR: hazard ratio; CI: confidence interval

If IL-1α has the potential to increase T cell anti-tumor responses, we proposed that increased circulating levels of IL-1α may represent a favorable anti-tumor immune response compared to lower IL-1α levels and would perhaps predict a more favorable response to cetuximab-based therapy - since T cell activity is an important mechanisms of action for cetuximab efficacy. We obtained pre-treatment serum samples from a cohort of 11 consented patients at the UIHC who were treated with cetuximab monotherapy or cetuximab-based chemotherapy (i.e. carboplatin, cisplatin, 5-FU, paclitaxel) and had available clinical outcome data. Analysis of IL-1α levels by ELISA revealed that IL-1α levels varied widely among the patients and ranged from undetectable (i.e. below limit of detection) to 418 pg/mL. Differences between pre-treatment IL-1α levels in patients with stable disease (SD, (*n* = 6)) compared to progressive disease (PD, (*n* = 5)) according to RECIST criteria were not significant (data not shown). There were also no differences in demographic or clinicopathological parameters between the 2 patient cohorts (Additional file 1: Table S1). None of the patients achieved a response of partial response (PR) or complete response (CR). Five of the patients had undetectable serum levels of IL-1α and 6 patients had detectable levels. Patients with detectable (n = 5) vs undetectable (n = 6) IL-1α levels were compared with time to progression. We found significantly longer PFS in patients with detectable IL-1α (median survival = 224 days) compared to undetectable (median survival = 132 days) IL-1α by ELISA (Fig. 7). These data suggest that circulating IL-1α levels may be promising as a predictive indicator of PFS in cetuximab-treated HNSCC patients and warrants further investigation in this area.

In summary our data indicate that cetuximab induces the secretion of pro-inflammatory cytokines which is triggered by the release of IL-1α and subsequent IL-1R1/MyD88-dependent signaling (Figs. 1, and 2). The release of IL-1α appears to be associated with an anti-tumor response since increased rIL-1α can induce tumor regression as a single agent in immunocompetent mice (Figs. 4B, and 5D) and enhance the anti-tumor activity of cetuximab (Fig. 5A). IL-1α-NPs were observed to be a relatively safe and effective option for IL-1α delivery (Fig. 5F) and the anti-tumor activity of the combination of cetuximab and IL-1α-NP was T-cell dependent (Fig. 6). Finally, detectable baseline serum levels of IL-1α were associated with significantly longer PFS in a limited cohort of R/M HNSCC patients treated with cetuximab-based chemotherapy compared to undetectable levels (Fig. 7). Altogether, the results presented here suggest that IL-1α in combination with cetuximab can induce a T cell-dependent anti-tumor immune response and may represent a novel immunotherapeutic strategy for EGFR-positive HNSCCs. This work also suggests that circulating IL-1α as a predictive biomarker for clinical outcomes to cetuximab-based therapy for HNSCC patients is worthy of further investigation.

## Discussion

The key findings of the data presented here center around the induction of an IL-1α/IL-1R/MyD88-dependent signaling pathway due to cetuximab treatment in HNSCC cells, and that IL-1α may be a promising immunotherapeutic strategy alone and in combination with cetuximab for the treatment of HNSCC. The ability of cetuximab to induce the secretion of pro-inflammatory cytokines such as IL-1α, IL-6 and IL-8 directly from HNSCC cells (Fig. 1) is supported by our previous work showing that a panel of EGFR inhibitors (i.e. cetuximab, panitumumab, erlotinib, lapatinib) increased the secretion of cytokines such as IL-4, IL-6, IL-8, GM-CSF and IFNγ [10]. The ability of cetuximab to induce the secretion of cytokines is consistent in both SQ20B and Cal-27 cell lines although variability in the extent of cytokine secretion is observed between experimental replicates (Fig. 1). This variability may be due to normal slight differences in EGFR ligand (EGF, TGFα) levels in serum-containing cell culture media [36] which can affect the efficacy of cetuximab binding to EGFR. Despite this variability, MyD88 and IL-1R1 knockdown was able to suppress cetuximab-induced cytokine production (Fig. 1D-I), and in some cases baseline cytokine levels confirming that IL-1R1/MyD88-dependent signaling is involved in cytokine production.

The results point to IL-1α as the ligand responsible for activation of the IL-1R1 since IL-1α but not IL-1β was detectable by ELISA after cetuximab treatment (Fig. 1A,D,G), and neutralization of IL-1α (but not IL-1β activity) significantly suppressed cetuximab-induced IL-6 secretion (Fig. 2B). Interestingly, administration of rIL-1α (but not rIL-1β) triggered the expression and secretion of IL-6 despite both ligands being able to bind and activate the IL-1R1 (Fig. 2C). The reason for this is unclear although expression of the soluble IL-1R2 (sIL-1R2) may be involved. The IL-1R2 functions as an IL-1 decoy receptor and is structurally similar to IL-1R1 except its cytoplasmic domain is truncated, and it lacks a TIR region rendering this receptor incapable of transmembrane signaling [37]. Membrane and soluble forms of the IL-1R2 exist and generation of sIL-1R2 can be due to matrix metalloproteinase cleavage of full-length membrane bound IL-1R2 into 45–47 kDa sIL-1R2 or by alternative splicing [37]. Prior studies have shown that sIL-1R2 binds to IL-1β with a higher affinity (10^− 10^ M) compared to IL-1α (10^− 8^ M) [38–40] and the IL-1β/sIL-1R2 disassociation rate is quite low and considered irreversible [41]. The preferential binding of sIL-1R2 to IL-1β prevents IL-1β from activating IL-1R1 which results in an underestimation of IL-1β concentrations by ELISA [41, 42]. Since cancer cells of epithelial origin can express IL-1R2 [43, 44], it is possible that in our case cetuximab/EGFR inhibition may be inducing the release of IL-1β as well as IL-1α but we are unable to detect IL-1β due to rapid binding by sIL-1R2. Blocking IL-1R2 expression may reveal detectable levels of IL-1β and is the subject of further studies in the context of cetuximab therapy.

IL-1 signaling has been reported in various studies (including our own) to be associated with poor prognosis due to the resulting downstream expression of genes involved in tumor progression including IL-6 and IL-8 [15, 45–48]. In contradiction to IL-1’s tumor-promoting role, IL-1 signaling has been shown to be involved in tumor cell killing via an anti-tumor immune response [49–51]. In order to begin to understand how IL-1 signaling may impact HNSCC tumor response to cetuximab, we showed that IL-1 blockade using anakinra did not enhance or affect tumor response to cetuximab treatment in both immunodeficient and immunocompetent mouse models (Fig. 2D-F) suggesting that under the described experimental conditions, IL-1 signaling may not play a major role in the anti-tumor efficacy of cetuximab. However, blocking IL-1 signaling with anakinra does have some limitations since it has a short half-life (median 5.7 h) necessitating daily dosing and 100–1000 fold excess of drug (in relation to IL-1 ligands) is required for appropriate blockade of IL-1 signaling [37]. Using the IL-1R1 knockdown SQ20B cells shown in Fig.S 1G-I in athymic nude mice, we showed that knocking down the IL-1R1 using both clones (shIL-1R#1 and shIL-1R#2) did not enhance but partially and significantly reversed the anti-tumor effect of cetuximab (Additional file 9: Figure S8). In these particular genetic IL-1R1 knockdown experiments we used a much higher dose of cetuximab (6 mg/kg) than the cetuximab dose (2 mg/kg) utilized for the SQ20B-xenograft mouse models described in the main manuscript, which caused a complete inhibition of tumor growth (Additional file 9: Figure S8A,B). Despite this high dose, IL-1R1 knockdown was able to partially reverse the effect of cetuximab (Additional file 9: Figure S8A,B) which altogether suggests that IL-1 blockade does not enhance cetuximab efficacy, but under certain conditions may be detrimental for optimal cetuximab efficacy.

The important role of IL-1 signaling in anti-tumor immune response [22] provided an opportunity to determine if an increase in IL-1 signaling would enhance the efficacy of cetuximab. IL-1 signaling has been proposed as a key mediator of host defense against malignancies through its role on NK cell activity (i.e. IFNγ production and ADCC) [49]. In fact, NK-cell activity can be significantly inhibited by anakinra (IL-1RA), or by neutralizing antibodies for IL-1 ligands [52]. Although we found that cetuximab in combination with increased IL-1α was effective in athymic nude mice (Figs. 3, and 4A) which are capable of robust NK cell responses, we found no evidence of NK cell involvement when looking at NK cell phenotypes in spleens and tumors from immunocompetent mice (Additional file 5: Figure S4, Additional file 6: Figure S5). IL-1 is also able to directly enhance survival of CD4+ T cells and induce secondary CD8+ T cell responses characterized by enhanced granzyme B expression and increased IFNγ production [53–55]. In support of this we observed increased levels of CD8+ T cells and decreased PD1 + CD4+ and CD25 + CD4+ T cells in spleens of BALB/c mice administered cetuximab+IL-1α-NP compared to control (Fig. 5G-I), and T cells appeared to be required for the anti-tumor mechanism of IL-1α in particular since rIL-1α (as a single agent) demonstrated significant anti-tumor activity in BALB/c mice (Fig. 4B) but not nude mice (Fig. 4A). Furthermore, depletion of CD4+ and CD8+ T cells significantly reversed the effect of cetuximab+IL-1α-NP (Fig. 6) suggesting that IL-1α in combination with cetuximab can induce a T cell-dependent anti-tumor immune response.

Based on the anti-tumor properties of IL-1 ligands, recombinant IL-1 ligands were previously pursued as anti-cancer agents. Clinical studies conducted the late 1980s and early 1990s have shown that recombinant IL-1α (marketed as Dainippon and Immunex) can be safely given to human cancer patients [56]. Unfortunately, dose-related side effects such as hypotension, fever, vomiting and abdominal pain although manageable, resulted in lessened enthusiasm for continued production of human rIL-1α for clinical trials. The dramatic weight loss, lethargy and diarrhea observed in mice during rIL-1α treatment supports these prior observations (Fig. 4C, D). In an attempt to reduce the development of side effects, we used amphiphilic polyanhydride copolymers based on CPTEG and CPH which have been reported to be excellent delivery systems for various payloads in oncology and immunology-based research [57–62]. CPTEG in the copolymer serves to maintain structural integrity and immunogenicity of the encapsulated immunogen [63]. The amount of CPH in the copolymer is associated with longer erosion kinetics [64, 65]. Modification of the monomers or molar ratios in the copolymer composition of polyanhydrides have been shown to alter the degradation rate thereby regulating the release kinetics of the payload [62, 65]. The IL-1α-NP formulation utilized in this work showed less than ideal release kinetics in vitro since the majority of the payload was released in the first 24 h and the remainder slowly released over the next 5 days (Additional file 4: Figure S3). Nevertheless, in vivo, we show remarkable tumor regression in 8 of 9 mice with a single administration of IL-1α-NPs in combination with cetuximab (Fig. 5E) with no obvious signs of toxicity (Fig. 5F) compared to the dramatic weight loss seen with soluble rIL-1α (Fig. 4C, D). The IL-1α-NP formulation as a single agent also triggered tumor regression, although in only 6 of the 10 mice in this treatment group (Fig. 5D), which explains the non-significance of this treatment group on average compared to the control (EMP-NP)-treated group (Fig. 5A). These results demonstrate the clear anti-tumor potential of IL-1α-NP and the release kinetics appeared to be sufficient to induce a safe and effective anti-tumor immune response and tumor regression. Further work is ongoing using additional delivery nanoparticle platforms for IL-1 immunotherapeutic strategies.

The role of IL-1α in anti-tumor response and the contribution to the remarkable tumor regression observed when in combination with cetuximab led us to inquire if increased circulating levels of IL-1α could serve as a predictive biomarker for favorable clinical outcomes in cetuximab-treated HNSCC patients. To date, there are no biomarkers used in clinical practice that can predict tumor response to cetuximab in HNSCC patients despite predictive biomarkers for response to EGFR inhibitors being well established in non-small cell lung cancer (NSCLC) (37) and colorectal cancer (CRC) (38). So far in an ongoing study, we have found clear evidence of significantly longer PFS in a small cohort (*n* = 11) of HNSCC patients (with available clinical outcome data) treated with cetuximab-based chemotherapy with detectable baseline IL-1α levels compared to undetectable baseline IL-1α by ELISA (Fig. 7), suggesting that IL-1α expression may be a predictive indicator of recurrence or PFS in cetuximab-based chemotherapy-treated HNSCC patients. Cetuximab is typically and routinely administered in combination with chemotherapy in R/M HNSCC patients as standard of care therefore it is likely that our findings will not be able to be validated in an appropriate homogeneous cetuximab-monotherapy-treated HNSCC patient cohort. The current findings are limited so far by the small number of patients, however our additional ongoing studies in cetuximab-based chemo/radiotherapy-treated HNSCC patient cohorts compared to non-cetuximab- treated patients from separate independent clinical trials should assist in validating these findings. Our preliminary findings of IL-1α as a biomarker for favorable outcome to cetuximab therapy contradicts conventional knowledge about IL-1α since IL-1 signaling is believed to be associated with poor survival outcomes and drug resistance [13, 14]. However we believe that the anti-tumor immune response associated with IL-1α/IL-1 signaling can promote an environment which would be beneficial for the success of select agents that trigger anti-tumor immune responses (including cetuximab, anti-PD1) and further studies are ongoing to investigate these ideas.

## Conclusions

Overall, immunotherapy is a strategy that is currently promising for HNSCC patients and based on recent clinical data with T-cell checkpoint inhibitors (anti-PD1) [66, 67], it is clear that promoting an active anti-tumor immune response can be highly therapeutic. This work highlights the possible clinical utility of IL-1α-NP as a safe and novel immunotherapeutic strategy as a single agent and for use in combination with cetuximab for HNSCC therapy. We believe that safely increasing IL-1 signaling in combination with cetuximab as a novel immunotherapeutic strategy would be promising in that we would be targeting both the tumor (via EGFR inhibition) resulting in high response rates, and the host innate and adaptive immune system (via increased T-cell responses) resulting in more durable tumor responses. This strategy along with IL-1α as a potential predictive biomarker, would represent a significant advancement in treatment options for R/M HNSCC patients where cetuximab-based chemotherapy remains the standard of care and possibly for other patients bearing EGFR-expressing tumors.

## Additional files

Additional file 1: **Table S1.** Characteristics of R/M HNSCC patients treated with cetuximab-based therapy. (DOCX 19 kb)

Additional file 2: **Figure S1.** Cal-27 tumor responses to anakinra differs by gender. (PPTX 113 kb)

Additional file 3: **Figure S2.** TUBO-EGFR tumor responses to cetuximab and IL-1 differs by gender. (PPTX 137 kb)

Additional file 4: **Figure S3.** Treatment with recombinant IL-1a triggers inflammation in the colonic mesentery. (PPTX 1628 kb)

Additional file 5: **Figure S4.** Characterization of 1.5% IL-1a 20:80 CPTEG:CPH nanoparticles. (PPTX 159 kb)

Additional file 6: **Figure S5.** Nanoparticle delivery of IL-1a in combination with cetuximab does not significantly affect NK cell levels in spleens. (PPTX 130 kb)

Additional file 7: **Figure S6.** Nanoparticle delivery of IL-1a in combination with cetuximab does not significantly affect K cell levels in tumors. (PPTX 150 kb)

Additional file 8: **Figure S7.** Nanoparticle delivery of IL-1a in combination with cetuximab does not significantly affect T cells levels in tumor. (PPTX 111 kb)

Additional file 9: **Figure S8.** Genetic knockdown of tumor IL-1R suppresses cetuximab efficacy. (PPTX 139 kb)

## Abbreviations

ADCC: antibody dependent cell cytotoxicity
ANA: anakinra
CTX: cetuximab
EGFR: epidermal growth factor receptor
HNSCC: head and neck squamous cell carcinoma
IL-1: Interleukin-1
IL-1RA: IL-1 receptor antagonist
IL-1α: Interleukin-1 alpha
IL-1β: Interleukin-1 beta
IL-6: Interleukin-6
IL-8: Interleukin-8
IRAK: IL-1 receptor-associated kinase
MMPs: matrix metalloproteinases
MyD88: myeloid differentiation primary response gene 88
NK cells: natural killer cells
PFS: progression free survival
R/M: recurrent and/or metastatic
TIR: toll-like-interleukin-1 receptor
