# Supplementary material for: Interleukin-1 alpha increases anti-tumor efficacy of cetuximab in head and neck squamous cell carcinoma
Source: J Immunother Cancer. 2019 Mar 19;7:79. doi: 10.1186/s40425-019-0550-z (PMC6425573; doi:10.1186/s40425-019-0550-z)
Supplement: Supplementary file 1 — Table S1. Characteristics of R/M HNSCC patients treated with cetuximab-based therapy. (DOCX 19 kb) [file 40425_2019_550_MOESM1_ESM.docx]

| **Patient characteristics** | ***Total***  ***n* = 11** | ***SD***  ***n* = 6** | ***PD***  ***n* = 5** | **p value** |
| --- | --- | --- | --- | --- |
| *Mean age (years) at diagnosis*  (range) | 53.5  (42-69) | 58.3  (43-69) | 47.6  (42-57) | p=0.058 |
| *Primary tumor site*  Oral cavity  Oropharynx  Larynx  Unspecified | 36% (4)  9% (1)  27% (3)  27% (3) | 33% (2)  17% (1)  17% (1)  33% (2) | 40% (2)  0% (0)  40% (2)  20% (1) | p=0.89 |
| *Location of recurrence/metastasis*  Local  Distant  Both | 64% (7)  27% (3)  9% (1) | 50% (3)  33% (2)  17% (1) | 80% (4)  20% (1)  0% (0) | p=0.49 |
| *Chemotherapy prior to cetuximab-based therapy*  Yes  No | 55% (6)  45% (5) | 67% (4)  33% (2) | 40% (2)  60% (3) | p=0.38 |
| *Radiation prior to cetuximab-based therapy*  Yes  No | 91% (10)  9% (1) | 100% (6)  0% (0) | 80% (4)  20% (1) | p=0.46 |
| *Surgery prior to cetuximab-based therapy*  Yes  No | 64% (7)  36% (4) | 67% (4)  33% (2) | 60% (3)  40% (2) | p=0.82 |
| *Radiation concurrent with cetuximab-based therapy*  Yes  No | 18% (2)  82% (9) | 17% (1)  83% (5) | 20% (1)  80% (4) | p=0.89 |
| *Chemotherapy concurrent with cetuximab-based therapy*  Yes  No | 82% (9)  18% (2) | 83% (5)  17% (1) | 80% (4)  20% (1) | p=0.89 |

**Supplementary Table 1. Characteristics of R/M HNSCC patients treated**

**with cetuximab-based therapy.**
