# Supplementary figures and images for: Interleukin-1 alpha increases anti-tumor efficacy of cetuximab in head and neck squamous cell carcinoma
Source: J Immunother Cancer. 2019 Mar 19;7:79. doi: 10.1186/s40425-019-0550-z (PMC6425573; doi:10.1186/s40425-019-0550-z)

## Slide 1
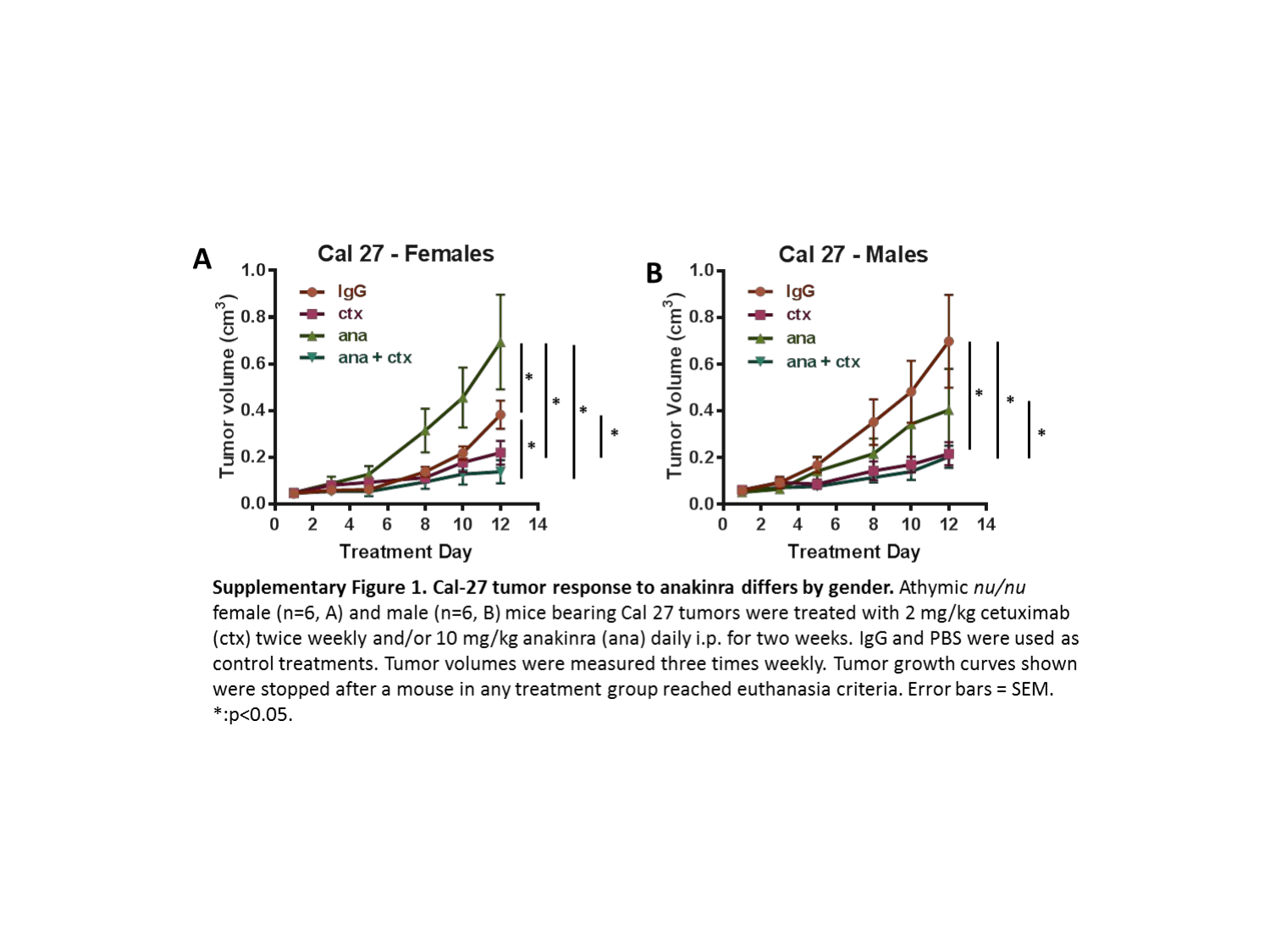

Supplement: Supplementary file 2 — Figure S1. Cal-27 tumor responses to anakinra differs by gender. (PPTX 113 kb) [file 40425_2019_550_MOESM2_ESM.pptx]

## Slide 1
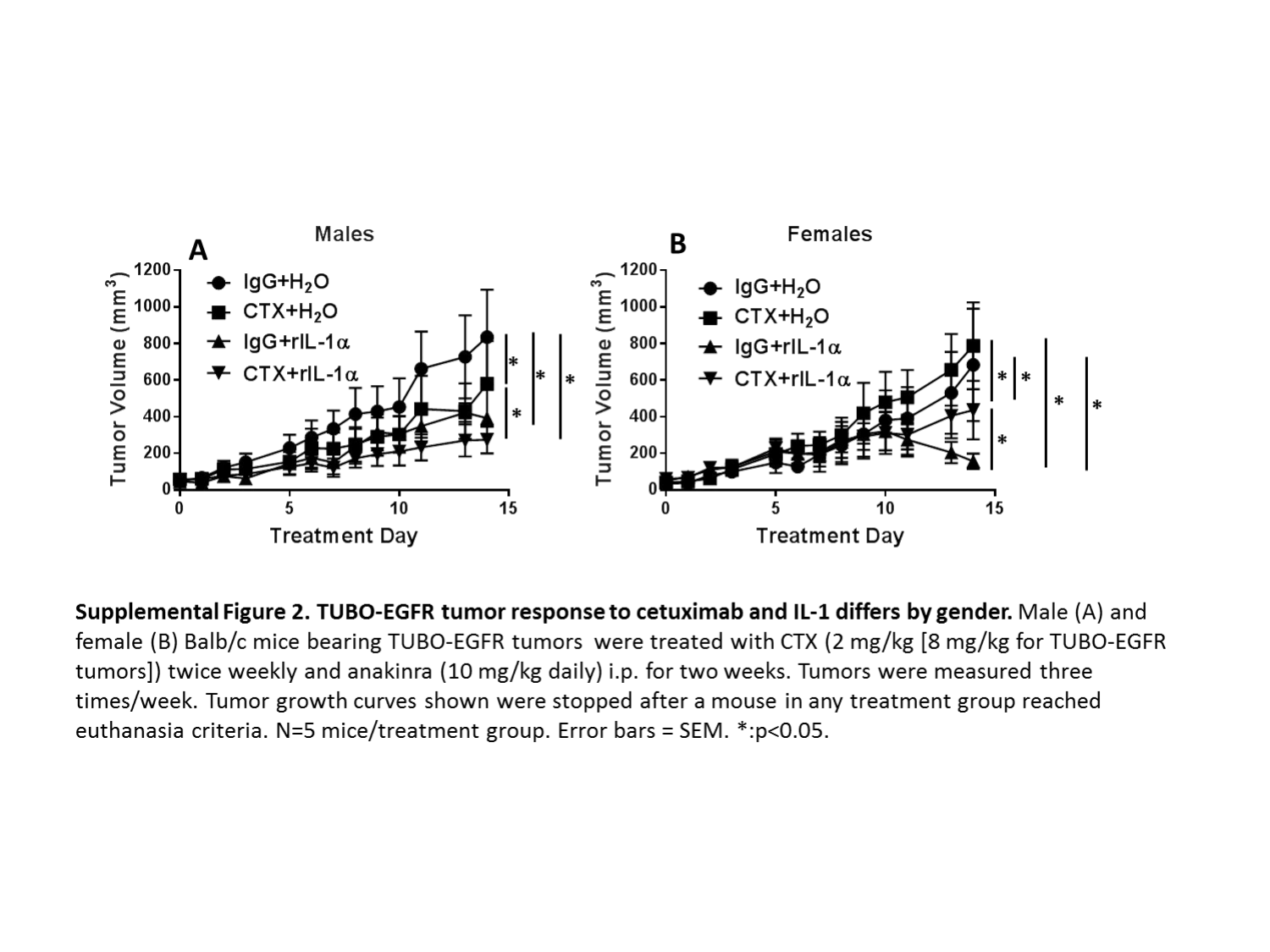

Supplement: Supplementary file 3 — Figure S2. TUBO-EGFR tumor responses to cetuximab and IL-1 differs by gender. (PPTX 137 kb) [file 40425_2019_550_MOESM3_ESM.pptx]

## Slide 1
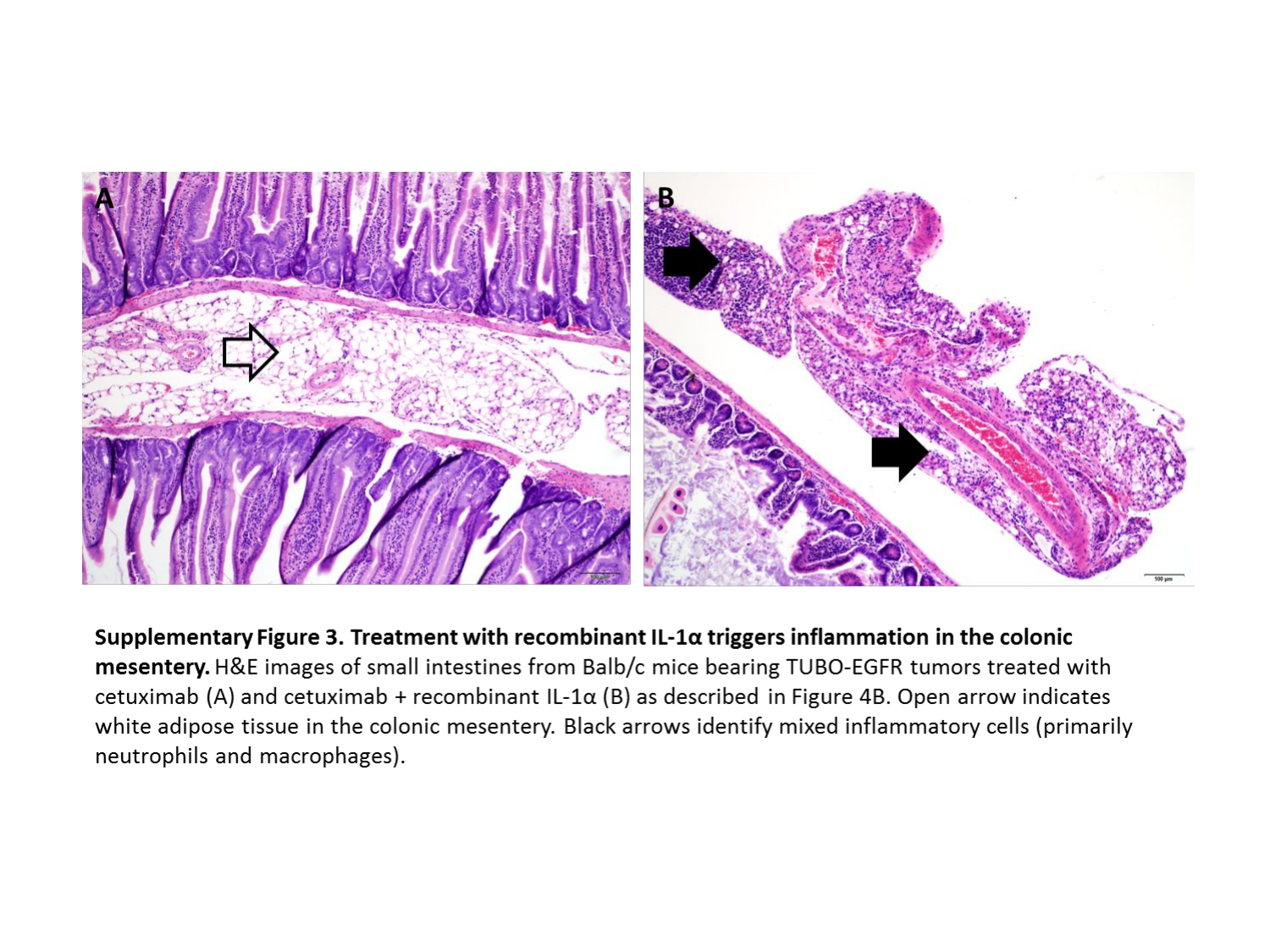

Supplement: Supplementary file 4 — Figure S3. Treatment with recombinant IL-1a triggers inflammation in the colonic mesentery. (PPTX 1628 kb) [file 40425_2019_550_MOESM4_ESM.pptx]

## Slide 1
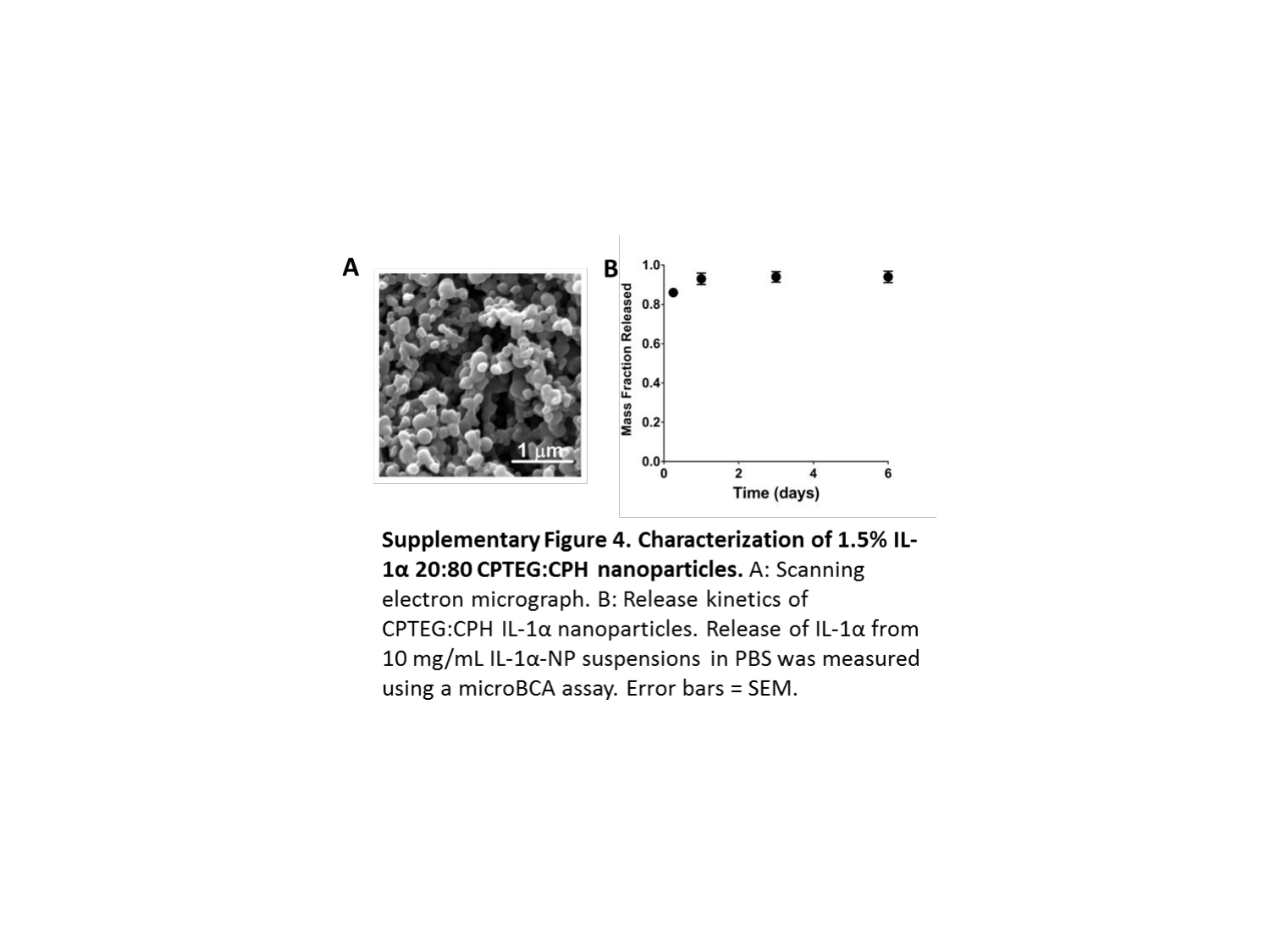

Supplement: Supplementary file 5 — Figure S4. Characterization of 1.5% IL-1a 20:80 CPTEG:CPH nanoparticles. (PPTX 159 kb) [file 40425_2019_550_MOESM5_ESM.pptx]

## Slide 1
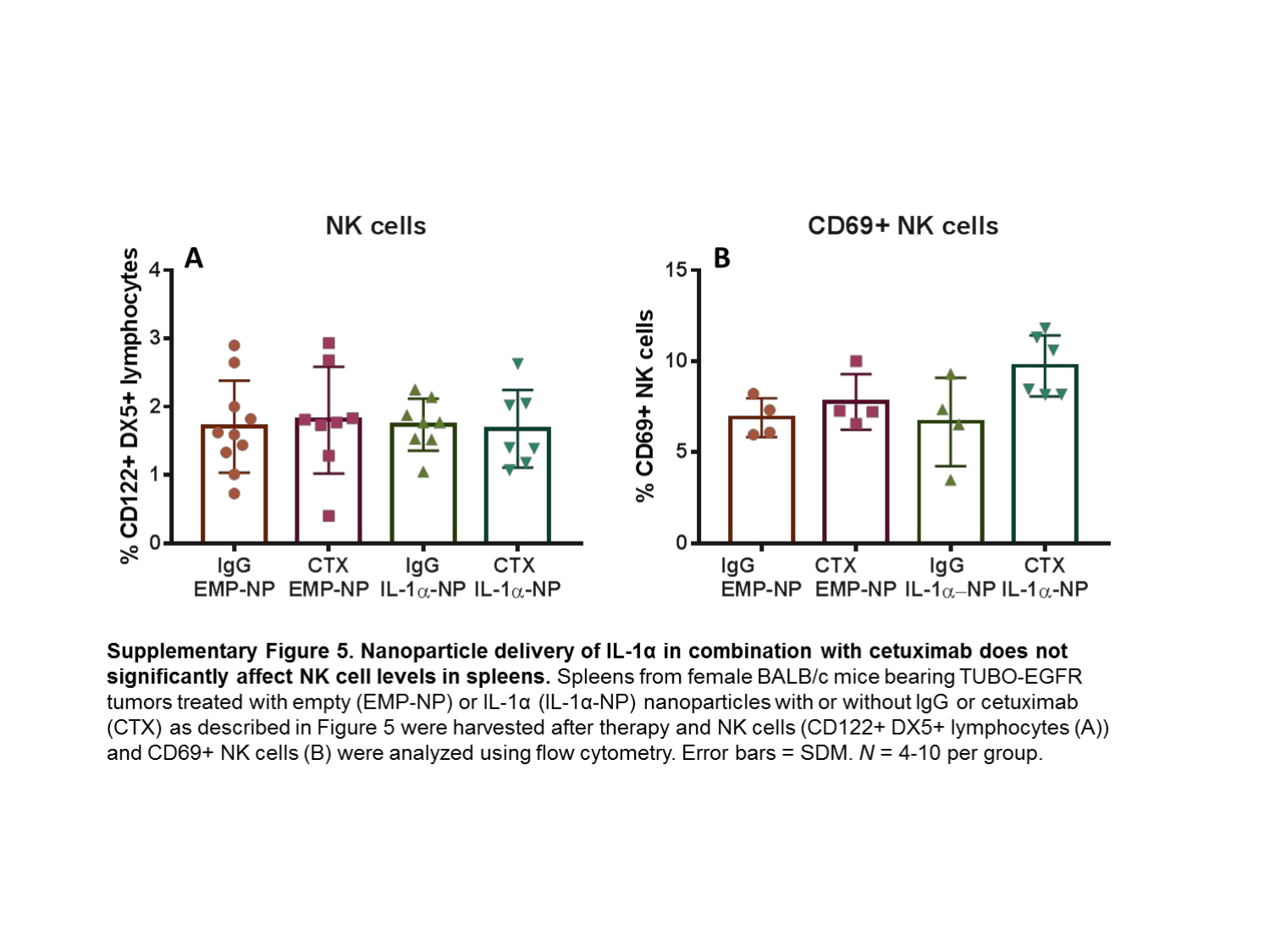

Supplement: Supplementary file 6 — Figure S5. Nanoparticle delivery of IL-1a in combination with cetuximab does not significantly affect NK cell levels in spleens. (PPTX 130 kb) [file 40425_2019_550_MOESM6_ESM.pptx]

## Slide 1
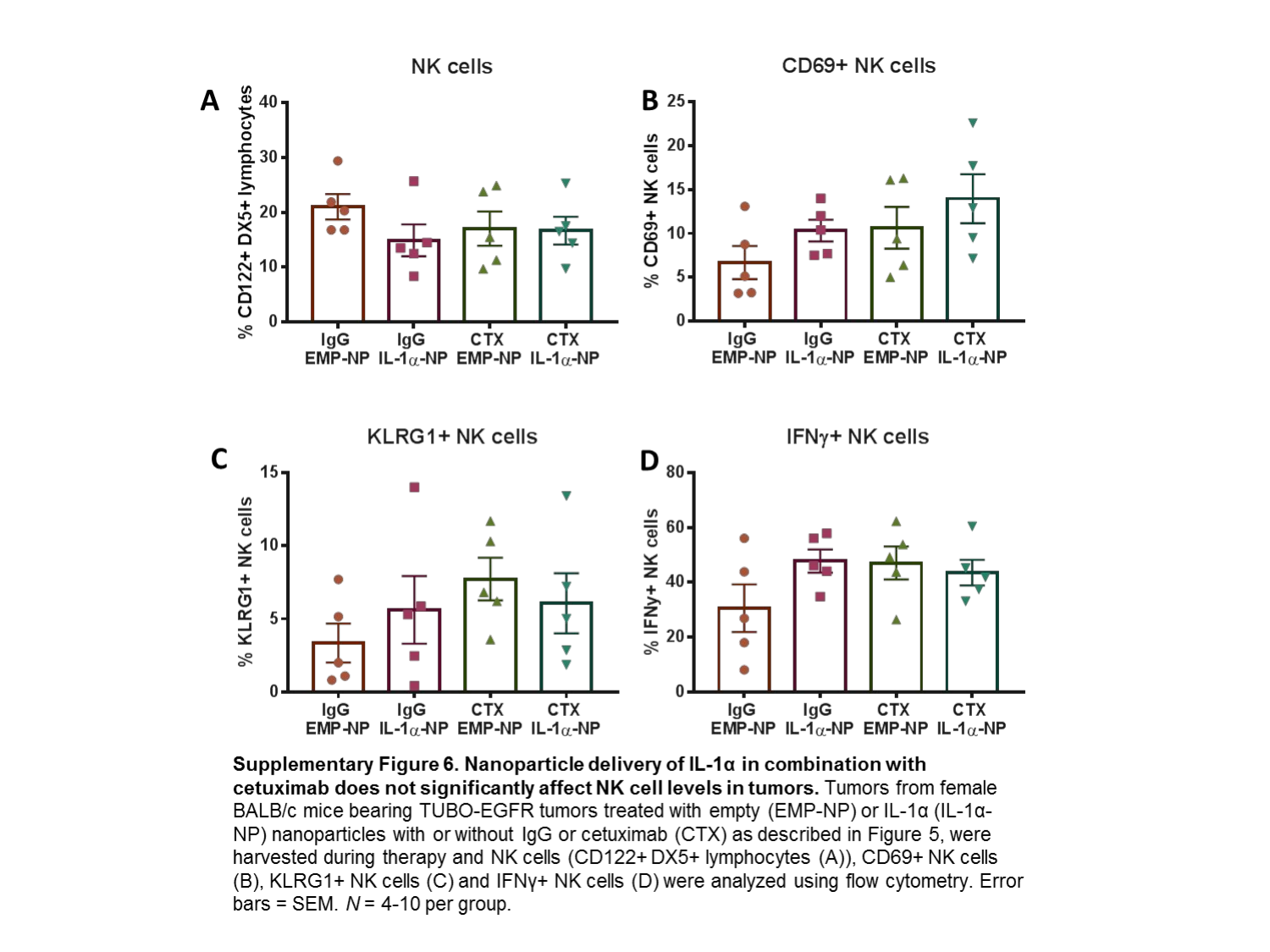

Supplement: Supplementary file 7 — Figure S6. Nanoparticle delivery of IL-1a in combination with cetuximab does not significantly affect K cell levels in tumors. (PPTX 150 kb) [file 40425_2019_550_MOESM7_ESM.pptx]

## Slide 1
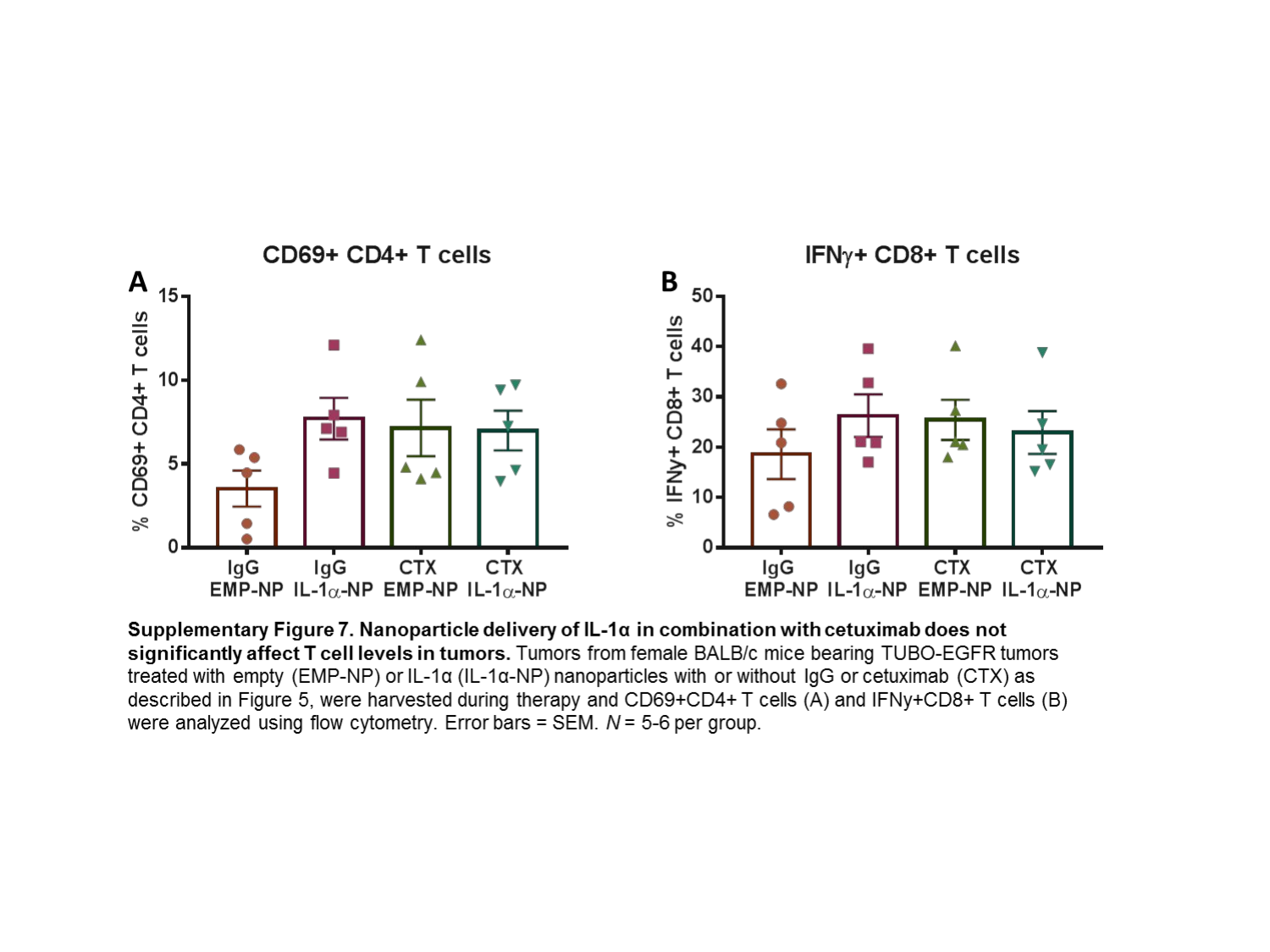

Supplement: Supplementary file 8 — Figure S7. Nanoparticle delivery of IL-1a in combination with cetuximab does not significantly affect T cells levels in tumor. (PPTX 111 kb) [file 40425_2019_550_MOESM8_ESM.pptx]

## Slide 1
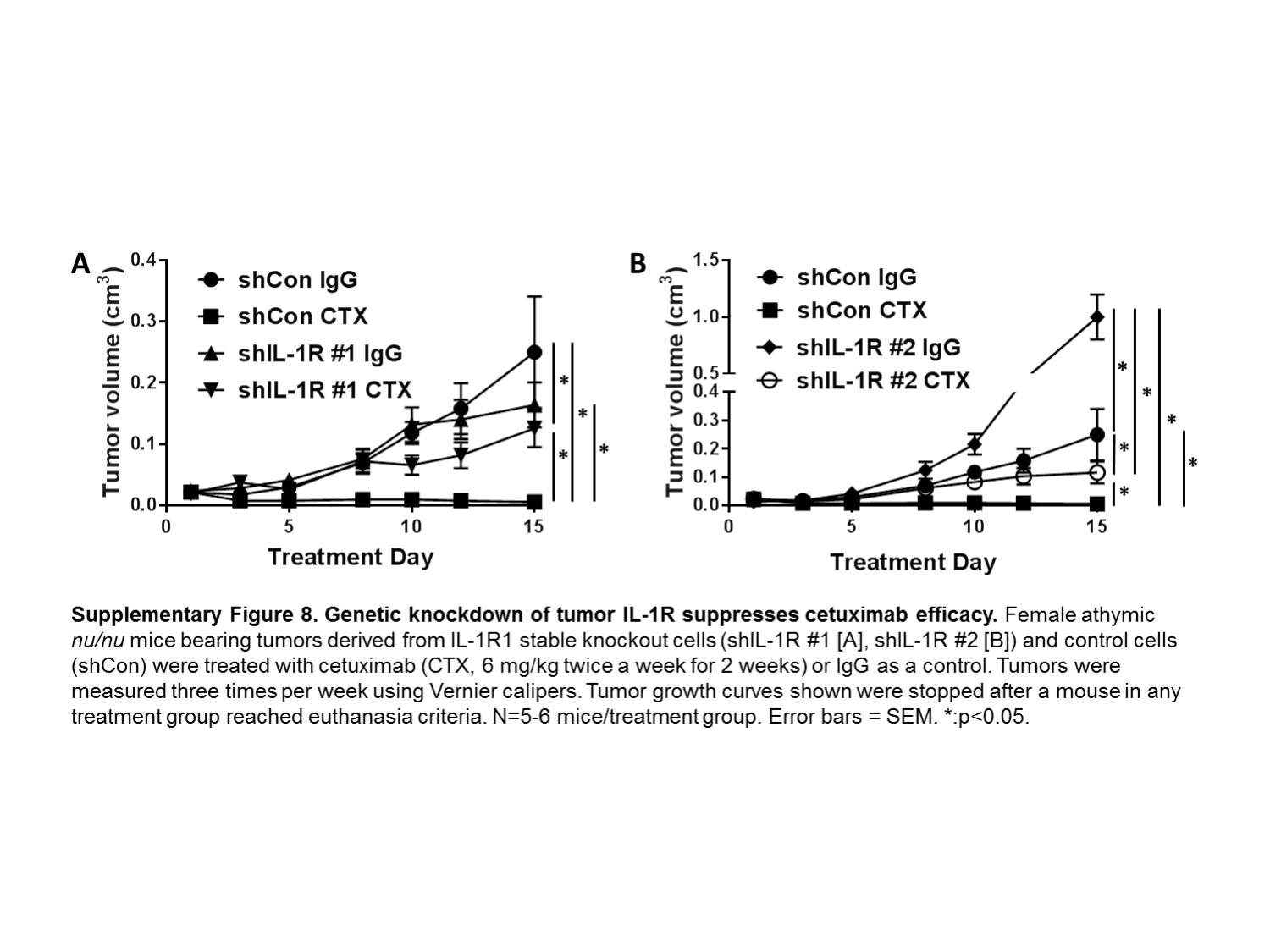

Supplement: Supplementary file 9 — Figure S8. Genetic knockdown of tumor IL-1R suppresses cetuximab efficacy. (PPTX 139 kb) [file 40425_2019_550_MOESM9_ESM.pptx]
